# New Fossil Megalopteran and Megalopteran-like Larvae, a First Extinct Larval Morphology of Megaloptera, and Possible Larvae of Myxophagan Beetles

**DOI:** 10.3390/insects17020197

**Published:** 2026-02-12

**Authors:** Corleone F. Stahlecker, Ana Zippel, Carolin Haug, Gideon T. Haug, Scott R. Anderson, Viktor Baranov, Pepe Knapp, Patrick Müller, Joachim T. Haug, Simon J. Linhart

**Affiliations:** 1 Biocenter, Ludwig-Maximilians-Universität München, Großhaderner Str. 2, 82152 Planegg-Martinsried, Germany; corleone.stahlecker@campus.lmu.de (C.F.S.); chaug@biologie.uni-muenchen.de (C.H.); jhaug@biologie.uni-muenchen.de (J.T.H.); 2 GeoBio-Center, Ludwig-Maximilians-Universität München, Richard-Wagner-Str. 10, 80333 München, Germany; 3 Fakultät für Biowissenschaften, Universität Heidelberg, Im Neuenheimer Feld 234, 69120 Heidelberg, Germany; gideon.haug@palaeo-evo-devo.info; 4 Independent Researcher, 609 Fieldstone Drive, Moon Township, PA 15108, USA; 5 Doñana Biological Station EBD-CSIC, 41092 Seville, Spain; 6 Independent Researcher, Kreuzbergstr. 90, 66482 Zweibrücken, Germany

**Keywords:** Myanmar amber, Grès à Voltzia, Baltic amber, Triassic, shape analysis

## Abstract

We report new fossil larval specimens that have characters of the group Megaloptera, i.e., are either megalopteran or megalopteran-like larvae from the Triassic, Cretaceous, or Eocene. Fossils from the latter two times are preserved in amber. One of the new fossils reveals that some earlier reported presumed megalopteran-like fossils, that have been already suspected to be beetles, are indeed more likely beetles of the group Myxophaga. A quantitative morphological comparison supports that most fossil megalopteran (and megalopteran-like) larvae did not differ significantly from extant megalopteran larvae, indicating no major losses of morphology over time. However, two newly reported fossil specimens from Kachin amber indeed separate from the other larvae, representing an extinct morphotype. The morphotype is characterised by a prominent terminal filament, indicating that the specimen is a representative of Sialidae. However, unlike most of the known larvae of this group, it possesses a rather elongate prothorax and strongly curved mandibles. These two characters are more commonly known in larvae of Corydalidae. It seems most likely that the presence of these characters in the new morphotype does not represent a plesisomorphy, but is more likely the product of convergent evolution.

## Simple Summary

We report new fossil larvae that have characteristics of Megaloptera, the group of dobsonflies, fishflies and alderflies. Older fossils from the Triassic are preserved as sedimentary fossils and younger ones from the Cretaceous and Eocene are preserved in amber. One of the new amber fossils shows more details than morphologically similar previously reported ones. The new details support the previous suspicion that the larvae are beetles, probably of the group Myxophaga. A statistical analysis was performed, indicating no large morphological losses over time. However, two newly reported larvae from amber have a today unknown morphology. The terminal filament indicates the larvae being representatives of Sialidae, alderflies. Untypical for the group is the elongated prothorax and strongly curved mandibles. This is more typical for Corydalidae, dobsonflies and fishflies. It is more likely that these characters are the results of convergent evolution instead of being ancestral characters.

## 1. Introduction

Holometabolans—beetles, bees, butterflies, mosquitoes, and their closer relatives—dominate our modern continental ecosystems. The group Megaloptera represents a comparably small ingroup of Holometabola, comprising only around 425 formally described species (extant and extinct) [1] (p. 4). Nonetheless, representatives of this group play an important role in our understanding of freshwater ecosystems, as their aquatic larvae serve as important predators in some freshwater communities and are used as indicators for ecosystem health [2]. In the extant fauna the group Megaloptera consists of the two sister groups Corydalidae and Sialidae. Adults of Corydalidae are either addressed as fishflies (ingroup Chauliodinae) or as dobsonflies (ingroup Corydalinae). The larvae of Corydalinae are known as hellgrammites. Adults of Sialidae are addressed as alderflies [2,3,4].

Typical for all life stages of megalopterans are a generally elongate body and a prognathous head with prominent mandibles. The aquatic larvae bear lateral filamentous structures along the abdomen, with one pair on each of the anterior 7–8 segments, in most cases assumed to serve as gills. In Corydalidae, larvae have typically eight pairs of lateral filaments, in Sialidae typically seven pairs of lateral filaments are present [5].

Morphological and ecological differentiation between immatures and adults is common for holometabolans [6] and is assumed to have played an important role in the current success of the group Holometabola [7]. An aquatic lifestyle for the immatures and a terrestrial lifestyle for the adults is a widespread strategy within Pterygota (“flying insects” [8]). Megalopterans represent a prime example for this strategy, as they spend the majority of their life span as aquatic larvae, then pupate on land and spend the last weeks of their life as winged, terrestrial adults [4].

The fossil record of Megaloptera is relatively scarce, even more so for the larvae [9]. Fossils that have been interpreted as Megaloptera are known from the Triassic (e.g., [10,11,12]), Jurassic (e.g., [13,14]), Cretaceous (e.g., [9,15]), Eocene (e.g., [16,17]) and Miocene [18,19]. Some of the older fossils of larvae, which show megalopteran-larva-like morphologies, such as *Srokalarva berthei* (Carboniferous), *Kargalarva permosialis* (Permian) and *Trialarva coburgensis* (Triassic), remain difficult to interpret in regards to their possible identity [9,20,21,22].

Here we report new Triassic sedimentary fossils from the Grès à Voltzia locality and new amber fossils of Cretaceous and Eocene age, which show megalopteran-larva-like morphologies and could represent fossil representatives of Megaloptera. We furthermore explore the morphological changes over geological time in a quantitative frame.

## 2. Material and Methods

### 2.1. Material

Nineteen new fossil specimens were investigated and documented for the present study. One of these new specimens is preserved in Baltic (Eocene) amber (PED 0529), seven are preserved in Kachin amber (Myanmar, Cretaceous [23,24,25]; BUB 5196, BUB 5220, BUB 5274, PED 1556, PED 1669, PED 2821, PED 4796), one is a sedimentary fossil from Liaoning Province (China, Cretaceous [26,27]; PED 4797), and ten are sedimentary fossils from the Grès à Voltzia Lagerstätte (France, Triassic [10,11,28]) (SMNS-p-75650-1 (149c_not_Diptera_larve), SMNS-P-75650-2 (149_not_Diptera), SMNS-P-75650-3 (149_III_AA_Megaloptera), SMNS-P-75650-4 (149_III_Aa_Megaloptera), SMNS-P-75650-5 (149_Megaloptera), SMNS-P-75650-6 (9449_233_8), SMNS-P-75650-7 (No#9_Megaloptera), SMNS-P-75650-8 (9357), SMNS-P-75650-9 (149b_not_Diptera), SMNS-P-75650-10 (9047_317)). At least one specimen (SMNS-P-75650-10) is from Bust (Bas-Rhin) [11]. The PED specimens are part of the Palaeo-Evo-Devo Research Group Collection of Arthropods, Ludwig-Maximilians-Universität München (PED), Germany. The BUB specimens are part of the collection of one of the authors (PM) and available after request. The SMNS specimens belong to the collection of the Natural History Museum Stuttgart (Staatliches Museum für Naturkunde Stuttgart, SMNS) and were part of the former Gall-Grauvogel collection.

Sixty-five extant specimens were newly documented and used in this study. They are preserved in ethanol and are part of the Zoological State Collection Munich (Zoologische Staatssammlung München, ZSM). These do not have accession numbers.

Additional 359 depicted megalopteran specimens from literature and online sources (bugguide.com) were used for the morphological analysis [1,5,9,12,13,14,15,16,17,18,21,22,29,30,31,32,33,34,35,36,37,38,39,40,41,42,43,44,45,46,47,48,49,50,51,52,53,54,55,56,57,58,59,60,61,62,63,64,65,66,67,68,69,70,71,72,73,74,75,76,77,78,79,80,81,82,83,84,85,86,87,88,89,90,91,92,93,94,95,96,97,98,99,100,101,102,103,104,105,106,107,108,109] (Supplementary Table S1). In total, a dataset of 437 megalopteran and megalopteran-like specimens (fossil and extant, adult and immature) was analysed. For comparison, a dataset of larvae of Gyrinidae (fossil and extant) was used from a previous study (see [110]: [17,47,63,64,97,111,112,113,114,115,116,117,118,119,120,121,122,123,124,125,126,127,128,129,130] (Supplementary Table S1). One of the new fossil larvae (BUB 5220) is interpreted as a larva of Gyrinidae and was also added to the previous dataset. In total, a number of 48 larval specimens of Gyrinidae could be included.

Specimen PED 4797, interpreted as a mayfly (Ephemeroptera), was not included in the statistical analysis. It is used as comparison material to demonstrate difficulties in the identification of sedimentary fossils.

### 2.2. Ethical Backgrounds

In recent years ethical concerns have been raised about working with Kachin amber from Myanmar, especially concerning so-called “parachute science” (e.g., [131,132]). While the situation is complicated due to the ongoing conflicts within the country, we are trying to establish collaborations with scientists in Myanmar [133,134] in order to resolve the situation and have built up an international network.

In the context of the here investigated fossils, we need to point out that also other conflict zones bear ethical issues. Parts of Ukraine are under Russian occupation since 2014. Fossils from these areas, deposited at the Paleontological Institute of the Russian Academy of Sciences in Moscow (PIN) and described by Russian scientists, may present an ethical concern. Although we do not imply that this indeed concerns the Ukrainian material included in this study, such ethical issues have, in our view, so far not been pointed out sufficiently (one example is Shevchuk et al. [135]) and are therefore emphasised here.

### 2.3. Documentation of Material

Ten Triassic specimens have been documented and were photographed using a Canon EOS Rebel T3i (Canon, Tokyo, Japan) equipped with an MP-E 65 mm objective (Canon, Tokyo, Japan) and a Canon MT-24EX Macro Twin Flash (Canon, Tokyo, Japan). To minimize reflections and improve the contrast between the fossils and the surrounding matrix, all specimens were photographed under cross-polarized light (cf. [136,137,138,139,140]). Measurements on these images were conducted with the program Fiji (open source).

Eight specimens (BUB 5196, BUB 5220, BUB 5274, PED 0529, PED 1556, PED 1669, PED 2821, PED 4797) were documented on a Keyence VHX-6000 digital microscope (Keyence, Osaka, Japan) at different magnifications, with different illumination settings (ring light, coaxial polarised light, transmitted light) and on different backgrounds (black, white, glass). The panorama function with multiple frames was used, each frame was stacked and fused, and the frames were stitched together with the built-in software of the microscope. The HDR function with multiple lightning was used to increase the contrast. A single amber specimen (PED 4796) was photographed with a variable zoom trinocular scope (1 to 4.5 base, with 10× WF and 20× lenses) and a Sony E995 camera (Sony, Tokyo, Japan). Images were further processed with the software Adobe Photoshop CS2 (Adobe, San José, CA, USA). Measuring of the specimens was done with the software ImageJ (version 1.54p; open source) or Inkscape (version 1.1; open source).

### 2.4. Shape Analysis

For our analyses, we examined a dataset comprising eight newly collected fossil specimens, 65 extant specimens from the ZSM, and 365 specimens sourced from the literature (see Supplementary Table S1). The body parts of interest were outlined in the digital drawing programs Inkscape and Affinity Designer (Canva, Sydney, Australia)**,** based on self-documented images or published figures. Only the better preserved half was drawn. Outline drawings were converted from PNG format to BMP or JPEG format using Adobe Photoshop CS2 or the R-statistical environment (version 4.4.2; open source; R Core Team 2025).

Depending on the availability and preservation of morphological features, sets of outlines were generated. Only specimens exhibiting correct orientation and minimal distortion were included in the analyses. Four body parts were considered: mandible, head capsule, prothorax, and the remaining trunk. With different combinations, ten distinct outline datasets were analysed, corresponding to the following anatomical configurations: (1) head capsule with mandibles, (2) single mandible, (3) head capsule, (4) whole body, (5) whole body without mandibles, (6) prothorax and head capsule with mandibles, (7) prothorax and head capsule, (8) prothorax, (9) trunk, and (10) trunk without prothorax.

Nine of these datasets were analysed using the SHAPE software suite (version 1.3; open source) [141] with 20 harmonics, while data set 03 (head capsule), was analysed using Momocs (open source) [142] implemented in the HaugShapeV2 package (open source) [143]. The resulting principal component (PC) matrices were subsequently plotted against each other to visualise the morphological variation within a multivariate morphospace. PC1 and PC2 were used as they explain most of the variation of the dataset.

## 3. Results

### 3.1. Description of Triassic Specimens

**Figure 1:**
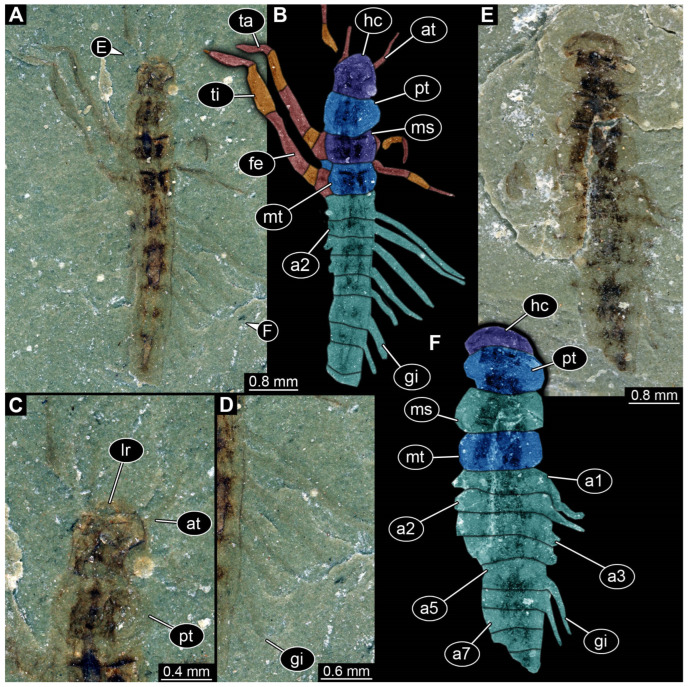
Specimens from Grèz à Voltzia. (**A**–**D**) Specimen 7531 (SMNS-P-75650-1). (**A**) Overview. (**B**) Overview, colour-marked. (**C**) Anterior part (head), detail. (**D**) Potential gills detail. (**E**,**F**) Specimen 7532 (SMNS-P-75650-2). (**E**) Overview. (**F**) Overview, colour-marked. Abbreviations: a1–7 = abdomen segments 1–7; at = antenna; fe = femur; gi = gill; hc = head capsule; ms = mesothorax; mt = metathorax; pt = prothorax; ta = tarsus; ti = tibia.

**Figure 2:**
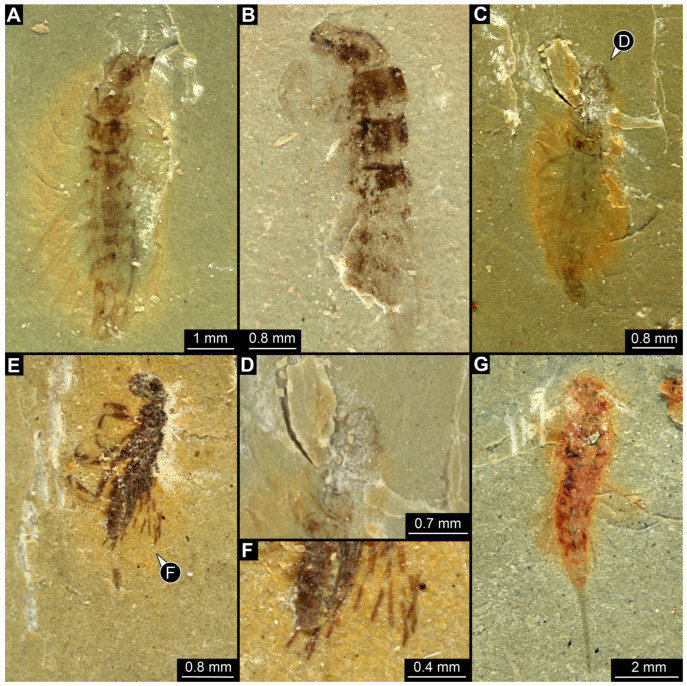
Specimens from Grèz à Voltzia. (**A**) Specimen 7533 (SMNS-P-75650-3), overview. (**B**) Specimen 7534 (SMNS-P-75650-4), overview. (**C**,**D**) Specimen 7535 (SMNS-P-75650-5). (**C**) Overview. (**D**) Anterior part (head), detail. (**E**,**F**) Specimen 7536 (SMNS-P-75650-6). (**E**) Overview. (**F**) Posterior part, detail. (**G**) Specimen 7537 (SMNS-P-75650-7), overview.

**Figure 3:**
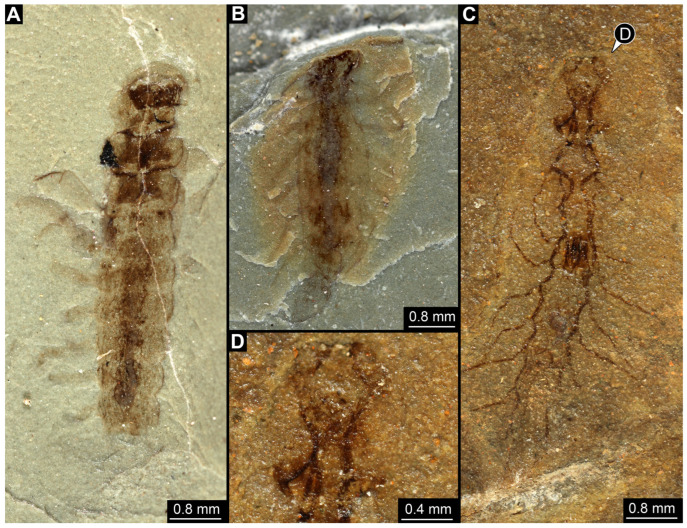
Specimens from Grèz à Voltzia. (**A**) Specimen 7538 (SMNS-P-75650-8), overview. (**B**) Specimen 7539 (SMNS-P-75650-9), overview. (**C,D**) Specimen 7540 (SMNS-P-75650-10). (**C**) Overview. (**D**) Anterior part (head), detail.

*SMNS-P-75650-1 (Figure 1A–D):* Single specimen preserved in dorsal view, with a body length of ca. 5.3 mm (Figure 1A). Head capsule about as long as wide (~0.6 mm; Figure 1C). Antennae present, as long as head capsule. Labrum vaguely discernible, but possibly an artefact due to poor preservation. Thorax with three segments (pro-, meso- and metathorax). All thorax segments discernible, appearing wider than long with the prothorax ca. 1.3× longer than the meso- and metathorax. Walking legs partly preserved, with remnants of the left walking leg of the prothorax present close to the left top of the head capsule. Left walking legs of meso- and metathorax appear as long as the thorax. Right walking legs only partly preserved. Lateral filamentous structures along abdomen, appear to get shorter from anterior to posterior (Figure 1D). Most anterior lateral filament on the right side appears of similar length to the walking legs of meso- and metathorax (~1.8 mm), while most posterior preserved lateral filament measures ~0.45 mm. Posterior end appears incomplete.
*SMNS-P-75650-2 (Figure 1E,F):* Single specimen preserved in dorsal view, with a body length of ca. 5.5 mm (Figure 1E). Head capsule appears partly concealed by prothorax, no antennae or mouthparts discernible. Thorax with three segments (pro-, meso- and metathorax). Thorax segments discernible, no walking legs preserved. Thorax segments all wider than long, of equal length (~0.6 mm) and appear to have tergites with round lateral edges. Abdomen segments with partly preserved lateral filamentous structures, most about as long as a thorax segment; the longest (abdomen segment 5) measuring ca. 0.8 mm. Posterior end appears incomplete.
*SMNS-P-75650-3 (Figure 2A):* Single specimen preserved in dorso-lateral view, with a body length of ca. 6.2 mm (Figure 2A). Head capsule as long as wide (~0.75 mm). Right antenna possible preserved, but not protruding the outline of head capsule. Right mandible discernible and appears forward protruding. Thorax with three segments (pro-, meso- and metathorax). All thorax segments discernible and wider than long of close to equal length, with the prothorax only slightly longer (~1.1×) than the meso- and metathorax. Lateral filamentous structures along abdomen, appear to get shorter from anterior to posterior, mostly preserved on the left body side. Most anterior lateral filament close to equal to the thorax length (~2 mm), while most posterior preserved filament measures ca. 0.5 mm. Posterior end possibly incomplete.
*SMNS-P-75650-4 (Figure 2B):* Single specimen preserved in dorso-lateral view, measuring ca. 4.3 mm (Figure 2B). Head oriented more in a lateral view, width not clearly discernible, length ca. 1.2 mm). Left antenna possible preserved, but not protruding outline of the head capsule. Left mandible discernible and appears forward protruding (~0.44 mm length). Thorax with three segments (pro-, meso- and metathorax). All thorax segments discernible and wider than long of close to equal length, with the prothorax only slightly longer (~1.1×) than the meso- and metathorax. Only one walking leg present, while all other walking legs missing; possibly coxae of second and third pair of walking legs present on the left side protruding posteriorly. Tarsus of preserved walking leg appears not subdivided. Few anterior abdomen segments preserved, appearing to bear lateral filamentous structures. Filaments of the first abdomen segment close to equal in length to the thorax (~2 mm). Posterior end of the abdomen clearly missing.
*SMNS-P-75650-5 (Figure 2C,D):* Single specimen preserved in dorsal view, with a body length of ca. 4.6 mm (Figure 2C). Head capsule slightly longer than wide, 1.15× (length ~0.73 mm). Labrum appears long and forward protruding (Figure 2D). Other mouthparts or antennae not discernible. Prothorax and anterior part of mesothorax not clearly discernible, metathorax discernible (length ~0.26 mm). Lateral filamentous structures along abdomen, appear to get shorter from anterior to posterior. Left filament of the first abdomen segment measures ca. 0.8 mm. Lateral filaments preserved on both sides but not always clearly discernible. Posterior end of the abdomen pointy in shape, possibly missing parts.
*SMNS-P-75650-6 (Figure 2E,F):* Single specimen preserved in lateral view, with a body length of ca. 4.4 mm (Figure 2E). Head capsule measures ca. 0.75 mm, width not discernible due to lateral preservation. Thorax segments not clearly discernible. All walking legs preserved, all appearing close to equal in size and measuring ca. 1.5 mm Lateral filamentous structures along abdomen, appear to get shorter from anterior to posterior. Lateral filament of the first abdomen segment measures ca. 1.5 mm, most posterior lateral filament measure ca. 0.2 mm. Posterior end not completely preserved with possible traces of a terminal unpaired filamentous structure (Figure 2F).
*SMNS-P-75650-7 (Figure 2G):* Single specimen preserved in dorsal or ventral view, with a body length of ca. 9.5 mm (Figure 2G). Anterior body region poorly preserved, head capsule and thorax segments not clearly discernible. Lateral filamentous structures along abdomen, appear to get shorter from anterior to posterior. Filament of the first abdomen segment measures ca. 1.8 mm, most posterior filament measures ca. 0.35 mm. Trunk end with a single median process oriented posteriorly (length ~2.9 mm).
*SMNS-P-75650-8 (Figure 3A):* Single specimen preserved in dorsal view, with a body length of ca. 5.8 mm (Figure 3A). Head capsule slightly wider than long, 1.2× (width ~0.9 mm). Thorax with three segments (pro-, meso- and metathorax) all wider than long, close to equal length, prothorax only slightly longer (~1.2×) than meso- and metathorax. First trunk tergite (pronotum) appears to have round lateral edges, tergites of meso- and metathorax appear more angular. Remnants of left second third walking legs appear to be present, but poorly preserved. Abdomen segments bear lateral filamentous structures, but poorly preserved. Longest visible lateral filament protrudes from the left side of abdomen segment 3 and measures ca. 1 mm. Posterior end of abdomen clearly missing.
*SMNS-P-75650-9 (Figure 3B):* Single specimen preserved in dorsal or ventral view, with a body length of ca. 4.8 mm (Figure 3B). Most of the anterior body region not preserved. Mesothorax partly preserved, metathorax present. Both appearing wider than long, tergites appear to have round edges. Lateral filamentous structures along abdomen, appear to get shorter from anterior to posterior. Abdomen appears wide in the middle, widest abdomen segment 4 (width ~1.25 mm). Left filament of the first abdominal segment represents the longest preserved filament with a length of ca. 1.6 mm; distal end curls back towards the body. Posterior end of abdomen possibly incomplete.
*SMNS-P-75650-10 (Figure 3C,D):* Single specimen preserved in dorsal or ventral view, with a body length of ca. 6.7 mm (Figure 3C). Unusual type of preservation with most structures discernible but only few remnants of the cuticle. Most of the visibly preserved structures appear to resemble the remnants of the trachea (compare discussion). Head capsule close to equal length to width ratio (length ~0.77 mm), but much slimmer in posterior region. No eyes, antennae or mouth parts discernible (Figure 3D). Remnants of thorax segments present but thorax segments not clearly distinguishable. Abdomen segments bear lateral filamentous structures. Right filament protruding from abdomen segment 2 appears to be longest present filament measuring ca. 1.4 mm in length. Posterior end possibly incomplete.

### 3.2. Description of Amber Specimens

**Figure 4:**
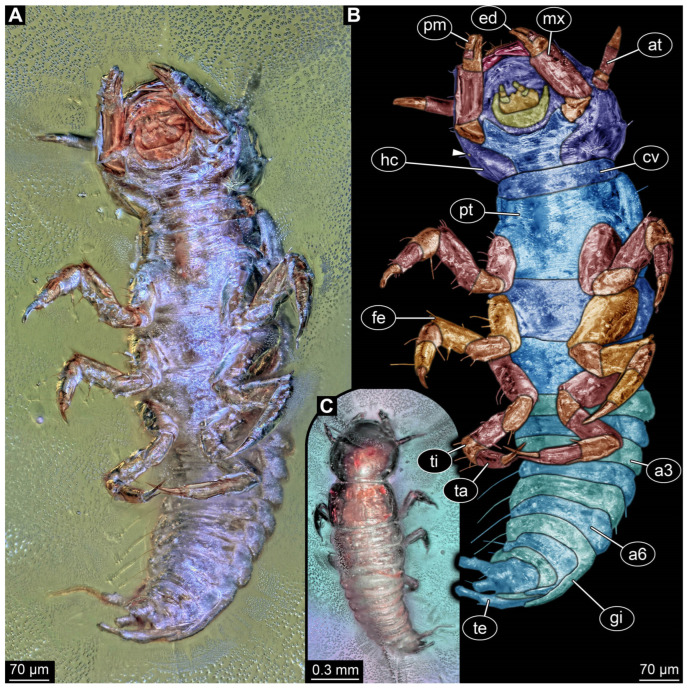
Specimen 7524 (PED 0529) from Baltic amber. (**A**) Ventral view. (**B**) Ventral view, colour-marked. (**C**) Dorsal view. Abbreviations: a3, 6 = abdomen segments 3, 6; at = antenna; cv = cervix; ed = endite; fe = femur; gi = gill; hc = head capsule; mx = maxilla; pm = palp of maxilla; pt = prothorax; ta = tarsus; te = trunk end; ti = tibia; arrow marks stemma.

*PED 0529 (Figure 4):* Single larval specimen preserved in Baltic amber. Specimen available in ventral (Figure 4A) and dorsal (Figure 4C) view. Total body length around 1 mm. Head capsule wider than long, 1.3× (width ~0.29 mm; Figure 4B). Possible single stemma discernible in ventral view (Figure 4B: arrow), several additional stemmata presumed. Both antennae discernible, with four elements each (Figure 4B). Antennae around 0.12 mm long, in total shorter than head capsule. Mouthparts partially accessible, directed anteriorly (Figure 4B,C): mandibles partially discernible with no additional teeth recognisable, maxillae longer than wide, 3.7× (at widest ~0.05 mm wide). Each maxilla with elongated stipes, longer than wide, endite and palp. Each palp with at least three elements (palpomeres). Labium semi-circular in ventral view, with indented ligula and a pair of palps with three elements each (palpomeres) of approximately same length.

All thorax segments (pro-, meso- and metathorax), wider than long, with mesothorax being the widest. Prothorax with cervical region anteriorly, whole segment including the cervical region wider than long, 1.4× (width ~0.27 mm). Mesothorax wider than long, 2.7× (width ~0.29 mm), metathorax wider than long. Each segment with a pair of long robust legs (length ~0.42 mm), each distally with a pair of claws. Abdomen segments 1–9 wider than long (width 0.09–0.22 mm), tapering posteriorly. Abdomen segment 7–9 with lateral sides drawn out into gills oriented posteriorly. Trunk end with two fleshy processes ventro-laterally and a single fleshy process dorso-medially. Head capsule and prothorax bear rare short hairs laterally. Legs with multiple short hairs throughout their length. Each side of abdomen segments with a single long hair laterally.

**Figure 5:**
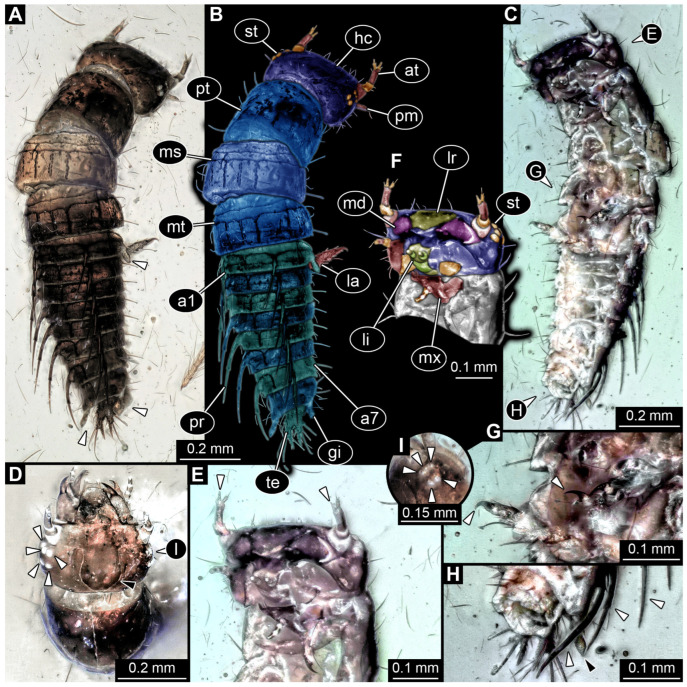
Specimen 7522 (PED 1556) from Kachin amber. (**A**) Dorsal view, arrows mark balloon-like gills. (**B**) Dorsal view, colour-marked. (**C**) Ventral view. (**D**) Dorsal view of head, white arrows mark stemmata, black arrow marks moulting suture. (**E**) Ventral view of head, arrows mark sensilla. (**F**) Ventral view of head, colour-marked. (**G**) Ventral view, legs detail, arrows mark claws. (**H**) Ventral view, posterior part, white arrows mark dorso-lateral protrusions, black arrow marks balloon-like gill. (**I**) Lateral view, eyehill detail, arrows mark stemmata. Abbreviations: a1, 7 = abdomen segments 1, 7; at = antenna; gi = gill; hc = head capsule; la = locomotory appendage (leg); li = ligula; lr = labrum; md = mandible; ms = mesothorax; mt = metathorax; mx = maxilla; pm = palp of maxilla; pr = protrusion; pt = prothorax; st = stemma; te = trunk end.3.3. Shape Analyses.

*PED 1556 (Figure 5):* Single larval specimen preserved in Kachin amber. Specimen available in dorsal (Figure 5A) and ventral (Figure 5C) view. Total body length around 1.35 mm. Head capsule wider than long, 1.1× (width ~0.33 mm (including labrum); Figure 5D), moulting structure on dorsal side discernible (Figure 5D: black arrow). Labrum drawn out anteriorly. Five stemmata discernible on both sides (Figure 5D,I: white arrows). Both antennae discernible, with four elements and sensory structure on the second distal element each (Figure 5E). Antennae around 0.14 mm long in total shorter than head capsule. Mouthparts accessible, directed anteriorly (Figure 5D): mandibles partly discernible with three teeth plus tip (Figure 5D), maxillae longer than broad, 1.8× (at widest ~0.05 mm width). Each maxilla with massive endite (longer and broader than palp) and palp (Figure 5E,F). Each endite with bended, tapering end and numerous (at least 4) setae distal, each palp with three elements (Figure 5E,F). Labium with ligula, prementum and mentum and with a pair of palps. Each palp with two elements (Figure 5E,F).

All thorax segments (pro-, meso- and metathorax), wider than long, with prothorax being the longest (length ~0.19 mm). Prothorax wider than long 1.5× (width ~0.28 mm), mesothorax wider than long 1.7× (width ~0.31 mm), metathorax wider than long 1.9× (width ~0.17 mm). Each segment with a pair of legs (length ~0.22 mm), each distally with a single claw (Figure 5G). Meso- and metathorax each with three node like structure orientated anterior-posterior. Eight abdomen segments and trunk end discernible (Figure 5A). Abdomen segments 1–7 each with a pair of dorso-lateral processes, processes slender with at least two recognisable unites (Figure 5A,C,H). Abdomen segments 1 and 8 with a pair of balloon/club-shaped lateral processes (possible gills; Figure 5A,H). Trunk end with two fleshy bifurcated processes orientated posteriorly (Figure 5A,H). Abdomen segments 1–8 with median located prominent setae (Figure 5A). Numerous long setae/hairs throughout the complete body (including the head). Legs with multiple short hairs throughout their length.

**Figure 6:**
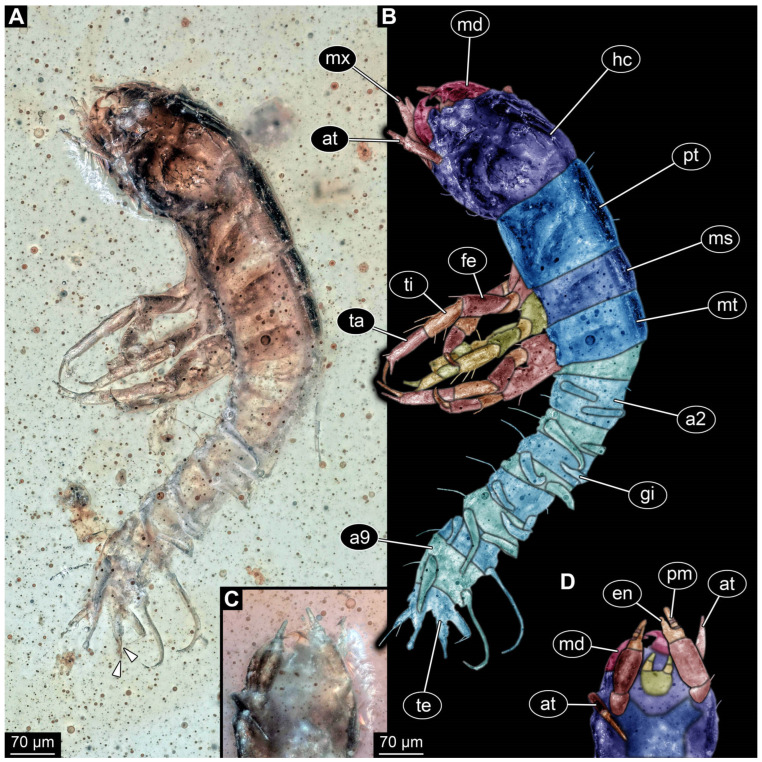
Specimen 7527 (PED 1669) from Kachin amber. (**A**) Dorso-lateral view. (**B**) Dorso-lateral view, colour-marked. (**C**) Ventral view of head. (**D**) Ventral view of head, colour-marked. Abbreviations: a2, 9 = abdomen segments 2, 9; at = antenna; ed = endite; fe = femur; gi = gill; hc = head capsule; md = mandible; ms = mesothorax; mt = metathorax; mx = maxilla; pm = palp of maxilla; pt = prothorax; ta = tarsus; te = trunk end; ti = tibia; arrows mark hook-shaped claws.

*PED 1669 (Figure 6):* Single larval specimen preserved in Kachin amber (Figure 6). Specimen available in dorso-lateral (Figure 6A) and head also in ventral (Figure 6C) view. Total body length around 1 mm. Head capsule as long as wide (width ~0.19 mm; Figure 6C,D), partially retracted under thorax (Figure 6B). Stemmata due to bubbles in amber not clearly discernible. Both antennae discernible, exact number of elements not discernible due to taphonomy (Figure 6B,D). Antennae around 0.11 mm long, in total shorter than head capsule. Mouthparts partially accessible (Figure 6B), directed anteriorly (Figure 6B): mandibles partially discernible, each with one tooth plus tip (Figure 6B), maxillae longer than wide, 3.6× (at widest ~0.04 mm wide). Each maxilla with elongated stipes, longer than wide, endite and palp. Each maxillary palp with at least four elements (palpomeres). Labium semi-circular in ventral view, without recognisable ligula and with a pair of palps with two elements each (palpomeres) of approximately same length.

Thorax segments (pro-, meso- and metathorax) only accessible in lateral view. Prothorax longer than meso- and metathorax (length ~0.14 mm). Meso- and metathorax approximately same length (length 0.07–0.08 mm). Each segment with a pair of long legs (length ~0.31 mm), each distally with a pair of large claws. Abdomen segments accessible in dorsal view, segments 1–9 wider than long (width 0.07–0.13 mm), tapering posteriorly. Abdomen segment 2–9 with lateral sides drawn out into gills oriented dorsally. Trunk end with two fleshy bifurcated processes orientated posteriorly. Head capsule and prothorax bear rare short hairs laterally. Legs with multiple short hairs throughout their length. Each side of abdomen segments with a single long hair laterally.

**Figure 7:**
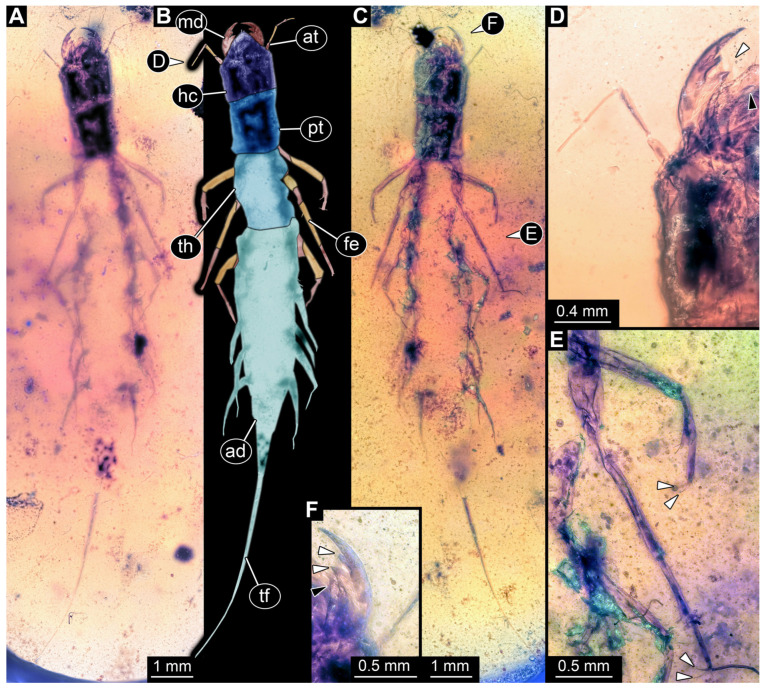
Specimen 7523 (PED 2821) from Kachin amber. (**A**) Dorsal view. (**B**) Dorsal view, colour-marked. (**C**) Ventral view. (**D**) Dorsal view of left half of the head, white arrow marks distal endite of maxilla, black arrow marks proximal endite of maxilla. (**E**) Legs detail, arrows mark claws. (**F**) Ventral view of mandible, white arrows mark distal teeth, black arrow marks possible third tooth. Abbreviations: ad = abdomen; at = antenna; fe = femur; hc = head capsule; md = mandible; pt = prothorax; tf = terminal filament; th = thorax.

*PED 2821 (Figure 7):* Single larval specimen preserved in Kachin amber. Specimen available in dorsal (Figure 7A) and ventral (Figure 7C) view. Total body length around 15.2 mm (including the terminal filament). Head capsule longer than wide, 1.3× (width ~1.25 mm; Figure 7C,D), labrum drawn out anteriorly, clypeo-labral structure discernible. Stemmata due to preservation not clearly discernible. Both antennae discernible, with four elements (Figure 7B,D). Antennae around 1.26 mm long, in total shorter than head capsule. Mouthparts partially accessible (Figure 7B), directed anteriorly (Figure 7B): mandibles partially discernible, at least one supposedly with three teeth plus tip (Figure 7F: arrows). Maxilla partially covered by syn-inclusions and other mouthparts, only partial maxillary palp and endite discernible (Figure 7C,D: arrow). Labium not discernible.

First thorax segment (prothorax) longer than wide, 1.3× (width ~1.1 mm). Separation between two other thorax segments (meso- and metathorax) not discernible. Each segment with a pair of long legs (length ~2.89 mm; Figure 7A–C,E), each distally with a pair of claws (Figure 7F: arrows). Separation between abdomen segments not discernible (at widest 1.62 mm wide), tapering posteriorly. Several abdomen segments with lateral sides drawn out into gills oriented posteriorly. Trunk end with a single median process orientated posteriorly (terminal filament), filament ~4.73 mm long. Head capsule bears anteriorly on labrum at least two short hairs. Legs with multiple short hairs throughout their length.

**Figure 8:**
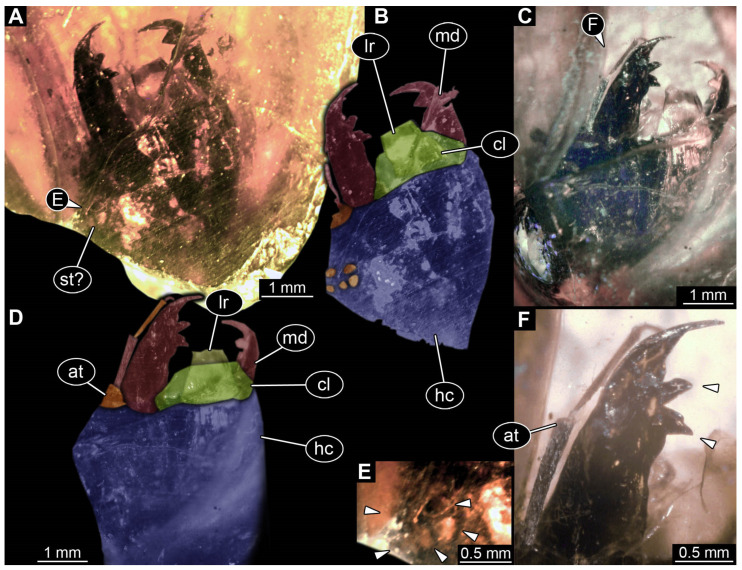
Specimen 7525 (PED 4796) from Kachin amber. (**A**) Dorsal view. (**B**) Dorsal view, colour-marked. (**C**) Dorso-lateral view. (**D**) Dorso-lateral view, colour-marked. (**E**) Eyehill detail, arrows mark possible stemmata. (**F**) Mandible and antenna detail, arrows mark teeth. Abbreviations: at = antenna; cl = clypeus; hc = head capsule; lr = labrum; md = mandible, st = stemma.

**Figure 9:**
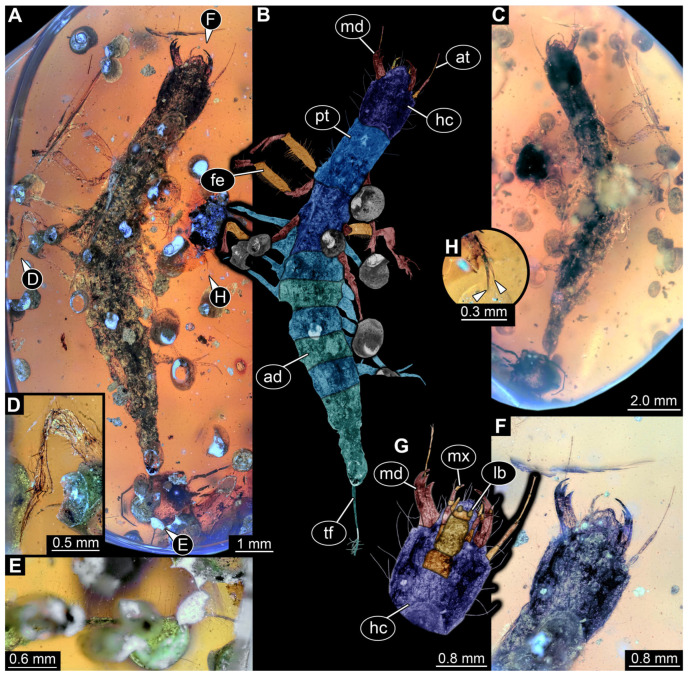
Specimen 7529 (BUB 5274) from Kachin amber. (**A**) Ventral view. (**B**) Ventral view, colour-marked. (**C**) Dorsal view. (**D**) Leg detail. (**E**) Terminal filament detail. (**F**) Ventral view of head detail. (**G**) Ventral view of head detail, colour-marked. (**H**) Claws detail. Abbreviations: ad = abdomen; at = antenna; fe = femur; hc = head capsule; lb = labium; md = mandible; mx = maxilla; tf = terminal filament; arrows mark claws.

*PED 4796 (Figure 8):* Single larval head preserved in Kachin amber. Specimen available in dorso-lateral view (Figure 8A,C). Head capsule longer than wide, 1.2× (width ~3.74 mm; Figure 8C,D), trapezoid-shaped labrum in dorsal view drawn out anteriorly, partially covered with semi-transparent clypeus (Figure 8B,D). Five ovaloid structures discernible laterally on the head capsule in dorso-lateral view (possibly stemmata; Figure 8A,B,E). Single antenna discernible, with four elements (Figure 8D,F). Antennae around 2.75 mm long, in total shorter than head capsule. Mouthparts partially accessible (Figure 8B), directed anteriorly (Figure 8B): large sclerotised mandibles discernible, each with three teeth (Figure 8F). Maxillae and labium not discernible.
*BUB 5274 (Figure 9):* Single larval specimen preserved in Kachin amber. Specimen available in dorsal (Figure 9A) and ventral (Figure 9C) view. Total body length around 13.5 mm (including the terminal filament). Head capsule longer than wide, 1.3× (width ~1.29 mm; Figure 9F,G), labrum drawn out anteriorly. Stemmata due to preservation not clearly discernible. Both antennae discernible, with four elements (Figure 9F,G). Antennae around 1.37 mm long, in total shorter than head capsule. Mouthparts partially accessible (Figure 9F,G), directed anteriorly (Figure 9F): mandibles discernible, each with two teeth (Figure 9F). Each maxilla with endite and palp. Each maxillary palp with four elements. Labium semi-circular in ventral view, prementum, mentum and ligula with a pair of palps, number of elements not discernible.

First thorax segment (prothorax) longer than wide,1.8× (width ~1.07 mm). Separation between two other thorax segments (meso- and metathorax) not discernible. Each segment with a pair of legs (length ~2.64 mm; Figure 9A–D), each distally with a pair of claws (Figure 9H: arrows). Separation between abdomen segments only visible until abdomen segment 5, wider than long (width up to 1.64 mm), tapering posteriorly. Several abdomen segments with lateral sides drawn out into supposed gills. Trunk end with a single median process orientated posteriorly (terminal filament), filament at least 1.81 mm long, with numerous hairs (setae; Figure 9E). Head capsule, labrum and prothorax bear numerous hairs (Figure 9A,F). Legs with multiple hairs throughout their length.

**Figure 10:**
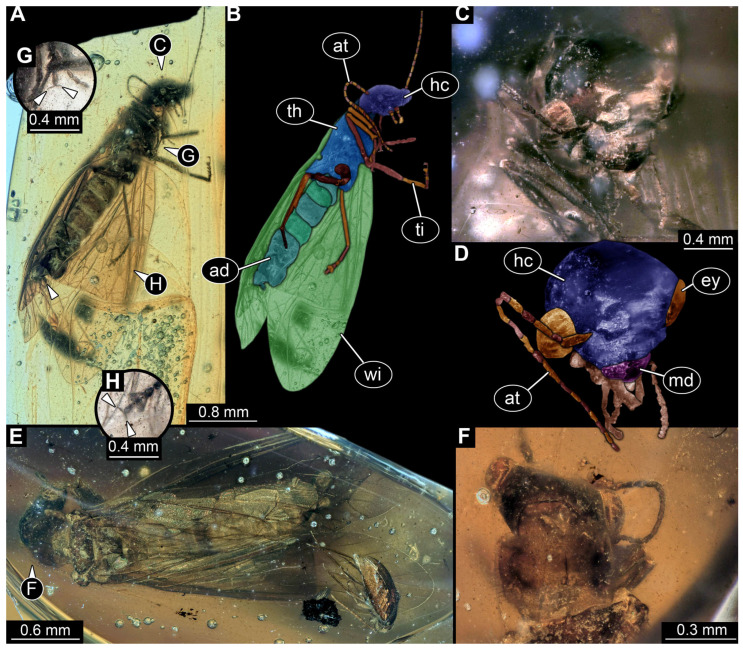
Specimen 7715 (BUB 5196) from Kachin amber. (**A**) Ventral view, arrow marks possible genitalia. (**B**) Ventral view, colour-marked. (**C**) Dorsal view of head. (**D**) Dorsal view of head, colour-marked. (**E**) Dorsal view of abdomen. (**F**) Dorsal view of prothorax. (**G**,**H**) Legs details, arrows mark claws. Abbreviations: ad = abdomen; at = antenna; ey = eye; hc = head capsule; md = mandible; th = thorax; ti = tibia; wi = wing.

*BUB 5196 (Figure 10):* Single adult specimen preserved in Kachin amber. Specimen available in ventro-lateral (Figure 10A) and dorsal (Figure 10E,F) view. Total body length around 2.79 mm (without wings). Head capsule wider than long, 1.3× (width including the eyes ~1.28 mm; Figure 10C,D). Two complex eyes are discernible. Both antennae discernible, with numerous elements (Figure 10A,B). Antennae around 2.5 mm long, in total longer than head capsule. Mouthparts partially accessible (Figure 10C,D): mandibles partially discernible, each with two teeth (Figure 10C,D). Maxilla partly discernible by maxillary palp, number of elements not discernible. Labium partly discernible.

First thorax segment (prothorax) wider than long, 1.2× (width ~0.48 mm) (Figure 10F). Separation between two other thorax segments (meso- and metathorax) not discernible. Meso- and Metathorax each bearing a pair of wings (Figure 10E). Each thorax segment with a pair of legs (length ~1.68 mm (hind leg); Figure 10A–D), each distally with a pair of claws (Figure 10G,H: arrows). Separation between abdomen segments only visible until abdomen segment 4, wider than long (width up to 0.34 mm).

**Figure 11:**
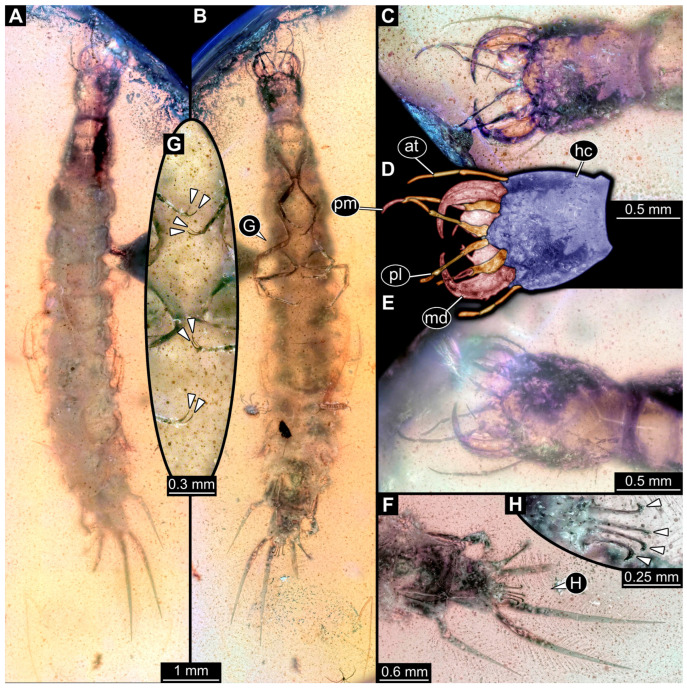
Specimen GR048 (BUB 5220) from Kachin amber. (**A**) Dorsal view. (**B**) Ventral view. (**C**) Ventral view of head. (**D**) Ventral view of head, colour-marked. (**E**) Dorsal view of head. (**F**) Ventral view of posterior part. (**G**) Legs detail, arrows mark claws. (**H**) Posterior part details, arrows mark hook-shaped claws. Abbreviations: at = antenna; hc = head capsule; pl = palp of labium; md = mandible; pm = palp of maxilla.

*BUB 5220 (Figure 11):* Single larval specimen preserved in Kachin amber. Specimen available in dorsal (Figure 11A) and ventral (Figure 11B) view. Prominent mouthparts are present: elongated antennae of three elements, bended toothless mandibles, elongated maxillary and labial palps. Maxilla with prominent proximal part and endite (Figure 11C–E). Three most anterior trunk segments (thorax) each with a pair of locomotory appendages (legs) (Figure 11B). Each leg bears two distal claws (Figure 11G). Posterior trunk segments (abdomen) with lateral “feathery” gills (Figure 11A,B,F). Posterior end of trunk with four hook-shaped protrusions (Figure 11H).

### 3.3. Description of Cretaceous Sedimentary Specimen

**Figure 12:**
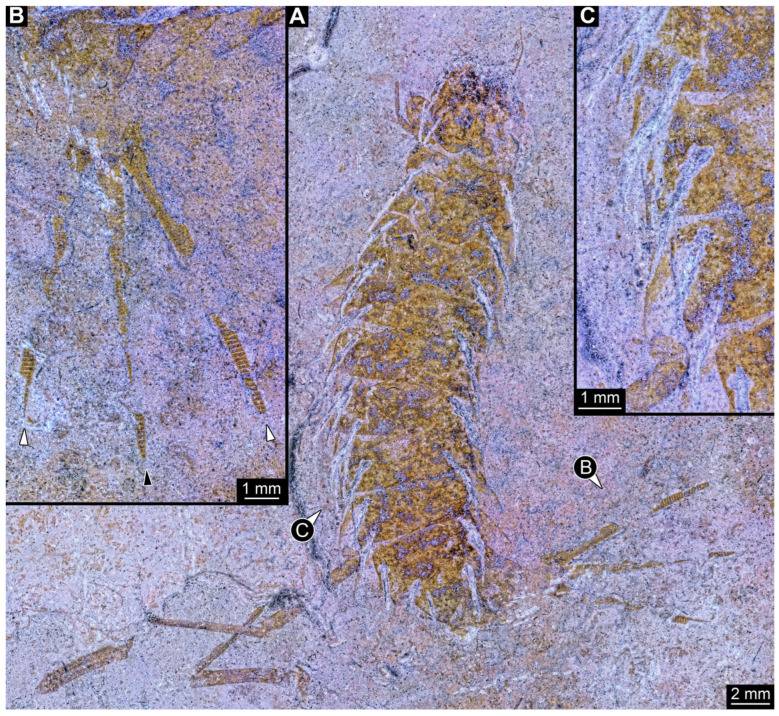
Specimen PED 4797 sedimentary fossil mayfly larva from Liaoning province (China). (**A**) Overview. (**B**) Posterior part, detail, white arrows mark possible cerci, black arrow marks possible terminal filament. (**C**) Lateral protrusions, detail.

*PED 4797 (Figure 12):* Single specimen preserved in dorsal or ventral view, with a body length of ca. 32.7 mm (Figure 12A). Head capsule present, eyes not discernible, antennae discernible above the head capsule. Mouthparts vaguely discernible but not clearly distinguishable. On the left side of the shown head capsule possibly the maxilla is discernible. Walking legs not present, possible remnants of the walking legs preserved on the bottom left of the trunk. Thorax tergites and abdomen tergites show lateral posteriorly bend extensions (Figure 12C). Lateral filamentous structures along trunk present (Figure 12C). At the posterior end cerci present (~8.5 mm long; Figure 12B) as well as one terminal unpaired filamentous structure (~8.5 mm long; Figure 12B), both cerci and terminal filamentous structure possibly distally incomplete.

### 3.4. Results of Shape Analyses

The detailed results of the different shape analyses can be found in Supplementary File S1.

*Analysis of head capsule and mandibles, without Gyrinidae:* The analysis resulted in six effective PCs together explaining over 91.3% of the overall variation. PC1 and PC2 together explain 73.1%. PC1 explains 57.0% of the overall variation. It describes the relative length of the labrum and the relative proximal width of the mandibles. Negative values indicate a proportionally long labrum and proximally slimmer mandibles, while positive values indicate a proportionally short labrum and proximally broader mandibles. PC2 explains 16.1% of the overall variation. It describes the distal width and relative length of the mandibles. Negative values indicate proportionally short and distally slim mandibles, while positive values indicate proportionally long and distally wide mandibles.
*Analysis of head capsule and mandibles, including larvae of Gyrinidae:* The analysis resulted in seven effective PCs together explaining over 92.7% of the overall variation. PC1 and PC2 together explain 74.0%. PC1 explains 49.1% of the overall variation. It describes the relative length of the labrum and the relative proximal width and curvature of the mandibles. Negative values indicate a proportionally long labrum and proximally narrower and straighter mandibles, while positive values indicate a proportionally short labrum and proximally broader and more curved mandibles. PC2 explains 24.9% of the overall variation. It describes the distal width and relative length of the mandibles. Negative values indicate proportionally long and distally broader mandibles, while positive values indicate proportionally short and distally narrower mandibles.
*Analysis of mandibles, without Gyrinidae:* The analysis resulted in seven effective PCs together explaining over 90.9% of the overall variation. PC1 and PC2 together explain 68.9%. PC1 explains 36.6% of the overall variation. It describes the curvature, distal width and dentition of the mandibles. Negative values indicate straight, distally slim, pointy mandibles with no dentition, while positive values indicate curved, distally broad mandibles with dentition. PC2 explains 32.3% of the overall variation. It describes the curvature and dentition mandibles. Negative values indicate highly curved mandibles with no dentition, while positive values indicate more straight mandibles with strong dentition.
*Analysis of head capsule, without Gyrinidae:* The analysis resulted in seven effective PCs together explaining over 92.6% of the overall variation. PC1 and PC2 together explain 66.4%. PC1 explains 42.0% of the overall variation. It describes the relative length and width of the labrum and relative width of the head capsule. Negative values indicate a proportionally short, broad labrum and a proportionally broad head capsule, while positive values indicate a proportionally long, slim labrum and a proportionally slim head capsule. PC2 explains 26.4% of the overall variation. It describes the relative length and width of the labrum and proportions of the head capsule. Negative values indicate a proportionally short, broad labrum and posteriorly broad head capsule, while positive values indicate a proportionally long, slim labrum and anteriorly broad head capsule.
*Analysis of whole body, without Gyrinidae:* The analysis resulted in eight effective PCs together explaining over 91.7% of the overall variation. PC1 and PC2 together explain 54.7%. PC1 explains 36.9% of the overall variation. It describes the relative length of the labrum and the relative width of the posterior body region. Negative values indicate a proportionally short labrum, while positive values indicate a proportionally long labrum. PC2 explains 17.8% of the overall variation. It describes the relative length of the labrum and the relative distal width of the mandibles. Negative values indicate a proportionally long labrum and distally broader mandibles, while positive values indicate a proportionally short labrum and distally narrower mandibles.
*Analysis of whole body, including larvae of Gyrinidae:* The analysis resulted in seven effective PCs together explaining over 89.8% of the overall variation. PC1 and PC2 together explain 70.7%. PC1 explains 47.6% of the overall variation. It describes the relative length of the labrum and the relative width of the mandibles. Negative values indicate a proportionally long labrum and proportionally broader mandibles, while positive values indicate a proportionally short labrum and proportionally narrower mandibles. PC2 explains 23.1% of the overall variation. It describes the relative length of the labrum and the curvature of the mandibles. Negative values indicate a proportionally short labrum and straighter mandibles, while positive values indicate a proportionally long labrum and more curved mandibles.
*Analysis of whole body without mandibles, without Gyrinidae:* The analysis resulted in six effective PCs together explaining over 89.1% of the overall variation. PC1 and PC2 together explain 64.7%. PC1 explains 51.3% of the overall variation. It describes the relative length and distal width of the labrum. Negative values indicate a proportionally short and distally broad labrum, while positive values indicate a proportionally long and distally narrow labrum. PC2 explains 13.5% of the overall variation. It describes the relative length and distal width of the labrum and the relative width of head capsule. Negative values indicate a proportionally long and distally narrow labrum and a proportionally narrower head capsule, while positive values indicate a proportionally short and distally broad labrum and a proportionally broader head capsule.
*Analysis of head capsule, mandibles and prothorax, without Gyrinidae:* The analysis resulted in seven effective PCs together explaining over 89.9% of the overall variation. PC1 and PC2 together explain 60.4%. PC1 explains 45.1% of the overall variation. It describes the relative length of the labrum, the relative proximal width of the mandibles and the relative width of the prothorax. Negative values indicate a proportionally short labrum, proximally broader mandibles and a proportionally narrow prothorax, while positive values indicate a proportionally long labrum, proximally narrower mandibles and a proportionally broad prothorax. PC2 explains 15.2% of the overall variation. It describes the relative length of the labrum and the relative length and width of the mandibles. Negative values indicate a proportionally short labrum and proportionally shorter and narrower mandibles, while positive values indicate a proportionally long labrum and proportionally longer and broader mandibles.
*Analysis of head capsule and prothorax, without Gyrinidae:* The analysis resulted in eight effective PCs together explaining over 90.0% of the overall variation. PC1 and PC2 together explain 51.3%. PC1 explains 32.6% of the overall variation. It describes the relative proximal width of the labrum and relative width of the clypeus and the proportions of the prothorax. Negative values indicate a proportionally proximally broad labrum and proportionally broad clypeus and a proportionally posteriorly broader prothorax, while positive values indicate a proportionally proximally narrow labrum and proportionally narrow clypeus and a proportionally anteriorly broader prothorax. PC2 explains 18.7% of the overall variation. It describes the relative length of the labrum, the proportions of the head capsule and relative width of the prothorax. Negative values indicate a proportionally short labrum, round head capsule and proportionally broad prothorax, while positive values indicate a proportionally long labrum, a proportionally posteriorly narrower head capsule and a proportionally narrow prothorax.
*Analysis of prothorax, without Gyrinidae:* The analysis resulted in four effective PCs together explaining over 93.6% of the overall variation. PC1 and PC2 together explain 82.9%. PC1 explains 60.4% of the overall variation. It describes the relative length and proportions of the prothorax. Negative values indicate a proportionally long prothorax that has a uniform width along the anterior-posterior body axis and is anteriorly curved, while positive values indicate a proportionally short prothorax that is proportionally anteriorly narrower and posteriorly broader. PC2 explains 22.5% of the overall variation. It describes the proportions of the prothorax antero-lateral and postero-lateral edges of the prothorax. Negative values indicate more angular antero-lateral and postero-lateral edges of the prothorax and a laterally more posteriorly curved anterior edge of the prothorax, while positive values indicate rounder antero-lateral and postero-lateral edges of the prothorax and a laterally more anteriorly curved anterior edge of the prothorax.
*Analysis of the trunk, without Gyrinidae:* The analysis resulted in seven effective PCs together explaining over 92.0% of the overall variation. PC1 and PC2 together explain 69.8%. PC1 explains 57.7% of the overall variation. It describes the relative width of the prothorax. Negative values indicate a proportionally narrower prothorax, while positive values indicate a proportionally broader prothorax. PC2 explains 12.1% of the overall variation. It describes how distinct the prothorax is set off from the mesothorax. Negative values indicate a more distinct set-off prothorax from the mesothorax, while positive values indicate a less distinct set-off prothorax from the mesothorax.
*Analysis of the trunk without prothorax, without Gyrinidae:* The analysis resulted in six effective PCs together explaining over 90.2% of the overall variation. PC1 and PC2 together explain 68.9%. PC1 explains 58.5% of the overall variation. It describes the relative width of meso- and metathorax and how distinct the abdomen segments are. Negative values indicate a proportionally narrow meso- and metathorax and no or less distinct abdomen segments, while positive values indicate a proportionally broad meso- and metathorax and strongly distinct abdominal segments. PC2 explains 10.3% of the overall variation. It describes the relative width of meso- and metathorax and the relative width of the posterior body region. Negative values indicate a proportionally broad meso- and metathorax and proportionally broad posterior body region, while positive values indicate a proportionally narrow meso- and metathorax and proportionally narrow posterior body region.

## 4. Discussion

### 4.1. Identity of Specimens

*Identity of new fossils: problems with the identification of fossil megalopteran-like larvae*

All of the here newly presented specimens share some morphological traits with extant megalopterans. In the case of the putative immature specimens [144,145] it must be stated that even in the extant fauna high degrees of convergence for some or many morphological traits are known in the larvae of certain ingroups of Coleoptera (see also dicusssion in [9]). Most notably, Hydrophilidae and Gyrinidae have larvae with striking similarities to megalopterans, e.g., aquatic lifestyle, an elongate body, forward-protruding prominent mandibles, and lateral filamentous structures along the abdomen [146,147].

Also larvae of Ephemeroptera, the group of mayflies, share many characters with larval megalopterans, as well demonstrated by PED 4797 (Figure 12). Mayfly larvae also have a terminal filament (Figure 12A) in addition to lateral filamentous structures that could serve as gills (Figure 12A,C; [148]). A terminal filament is already present in the ground pattern of Ectognatha [6] and occurs in mayfly larvae in combination with lateral gills. However, lateral structures could alternatively represent prominent styli as present in the ground pattern of Ectognatha and retained in Archaeognatha and Zygentoma. However, in all these a pair of prominent cerci is present, a clear difference to Megaloptera. Yet, there are examples of early branching lineages in Ectognatha in which possible cerci are rather short and could be overlooked or not preserved [149,150]

A consequence of the high convergence between all these groups and megalopterans is that poorly preserved specimens, and in some cases even well-preserved specimens that appear megalopteran-like, are not clearly identifiable as either megalopterans or representatives of the respective coleopteran ingroups. Therefore, the exact identification of these ambiguous fossils as representatives of specific holometabolan ingroups is often not possible, contrary to some statements in the current literature [21,22].

*Identity of Triassic sedimentary fossils*

The lack of the posterior end in megalopteran-larva-like specimens heavily restricts a reliable identification, an issue present in most of the new Triassic sedimentary fossils (compare Figure 1A,B, Figure 2A,B and Figure 3A,C). The lack of the posterior end, in addition to many other structures being not preserved well enough to make detailed interpretations for eyes, antennae, mouthparts, and walking legs, prohibits an exact identification for almost all of these specimens. In the case of SMNS-P-75650-7, however, the posterior end appears to be preserved more completely, also with a terminal filament (Figure 2G). As the terminal filament is present in the ground pattern of Ectognatha, but is lost in the ground pattern of Neoptera [150], and in combination with the lack of visible cerci and the overall habitus in SMNS-P-75650-7, Sialidae is the most plausible interpretation of this specimen. A similar but more ambiguous case represents SMNS-P-75650-6 as the posterior body region appears to not be completely preserved, but there are traces of a terminal filament present (Figure 2E). The argument is much weaker here, as it is not clearly discernible if cerci are present or not. Also the overall habitus differs from most of the other megalopteran-larva-like fossils from the same locality, including the possible representative of Sialidae. The most unusual here described fossil is SMNS-P-75650-10, as most of the outer cuticle is not preserved but putative remnants of the respiratory system are present (Figure 3C,D). The same fossil was already documented by Marchal-Papier [11] and is referred to as exuvia. Although the specimen is preserved in an unusual way, there is no clear indication that it is indeed an exuvia. A highly similar type of preservation has been described as *Izyumochauliodes aristovi* by Prokin & Bashkuev [12], also for putative fossil representatives of Megaloptera from Ukraine. As the posterior end of SMNS-P-75650-10 is not preserved, its exact identity remains ambiguous.

*Identity of specimens PED 0529 and PED 1669*

Specimens PED 0529 and PED 1669 show an overall similar morphology and various characters typical for larvae of Megaloptera; due to their similarities they are discussed together, although they originate from different types of ambers and different geological times. One character is the presence of lateral filamentous structures along the abdomen (Figure 4A,B and Figure 6A,B), as present in extant larval representatives of Megaloptera [5]. However, there are also many other groups of Insecta with lateral filamentous structures along the abdomen (see above), and the structures are better apparent towards the posterior end in the fossils.

A minor point is the presence of the tarsus with two claws (Figure 4A,B and Figure 6A,B), which at least makes an alternative interpretation as a polyphagan beetle unlikely. The trunk end, even though not well accessible, is also megalopteran-like. Specimen PED 1669 shows a pair of hook-shaped claws, at least on one side on the terminal end (Figure 6A). This morphology at the terminal end is typical for larvae of Corydalidae [5,32] (figure 75), [151] (figures 4C and 5C).

The mouthparts are similar to those of modern representatives of Corydalidae. The labrum and mandible are only partly accessible in both specimens. The accessible parts of the labrum seem to be shorter and not as exposed and forward-projecting (Figure 4C and Figure 6A) as in representatives of Corydalinae (hellgrammites) and are more similar to the labrum present in extant representatives of Chauliodinae [32]. The sensory region of the antennae is not accessible. The antennae consist of four elements, indicating that the specimen might be an early representative of Chauliodinae [96]. The mandibles of at least PED 1669 show teeth, that seem to be not as regular as in many representatives of Sialidae, cf. [5] (figure 68 right), [36] (figures 1–4 and 9), [58] (figure 10A), [68] (figure 7B), [70] (figure 23), [82] (figure 6), [95] (figures 16, 25, 36, 45, 54, 65, 75, 86, 91 and 96E), [100] (figure 9D), [151] (figure 7A), but more uneven and sharpened like in representatives of Corydalidae [32,96], cf. [36] (figures 58, 61, 73, 84, 86, 107 and 109), [151] (figures 4 and 5). This further supports the specimens being representatives of Corydalidae. Especially the maxillae have an appearance similar to those of modern representatives of Corydalidae. The cardo seems to be triangular, the stipes has a massive and elongate (cylindrical) shape, endite and palp of maxillae are clearly shorter than the stipes (Figure 4A,B and Figure 6C,D [58,151]).

Both specimens are rather small. This indicates that both are early stage larvae. A very similar appearing specimen has been reported from Taymyr amber [15] (figure 6) and has also been interpreted as an early stage larva of Megaloptera.

*Identity of specimen PED 4796*

In specimen PED 4796 at least five ovaloid structures probably representing stemmata are recognisable, which make an identification as a head of a holometabolan larva likely. Yet, due to limitations of the preservation the structures cannot be identified as stemmata with confidence. The morphology of the accessible head appendages (labrum, antenna, mandibles) shows similarities to those of modern larvae of Corydalidae. The mandibles are rather long and have two teeth plus tip each (Figure 8F). The mandibles being almost as long as the head capsule with sharpened teeth have been described for larvae of Megaloptera [96]. The antenna of larvae of Corydalinae (hellgrammites) has been described as consisting of five elements [60,96]. The exact number of elements in the antennae of the specimen cannot be identified, because the distal part is not accessible (Figure 8C,D,F). The area of the sensory structures on the secondmost proximal element of the antenna is unfortunately not accessible. This could show a further difference between the groups of Corydalinae and Chauliodinae [96]. Labrum and clypeus are well differentiated (Figure 8A–D). The arrangement is very similar to those of modern larvae of Corydalinae (hellgrammites; compare: [91] (figure 10), [152] (figure 1). An additional indicator for the identity is the relatively large size of the head capsule, about 5 mm including the labrum; this indicates a total size of around 35 mm. Extant larvae of Corydalidae reach a size from 20 to 80 mm [96]. Hence, also the size indicates that the specimen is a representative of Corydalidae.

*Identity of specimens PED 2821 and BUB 5274*

Specimens PED 2821 and BUB 5274 show an overall similar morphology and various characters typical for Megaloptera; due to their similarities they are discussed together. Similar to specimens PED 0529 and PED 1669 they have lateral filamentous structures along the abdomen (Figure 7A–C and Figure 9A–C) and a tarsus with two claws (Figure 7E and Figure 9H). A differing character is the long terminal filament (Figure 7A and Figure 9E). This is present in extant representatives of Sialidae and often bears numerous setae [5]. Setae can also be observed in specimen BUB 5274 (Figure 9E). The missing of setae at the terminal filament in specimen PED 2821 can also be due to taphonomy, the body posterior to the prothorax seems to be poorly preserved and partially dissolved (Figure 7A). In BUB 5274 the posterior part of the abdomen apears narrower as in modern counterparts. This is probably due to taphonomy. The prothorax of both specimens is untypical for modern representatives of Sialidae, by being longer than broad. For modern representatives of Sialidae, the prothorax is wider than long, at least for adults [80]. A wider than long prothorax for the whole group Sialidae is also supported by the quantitative analysis of the prothorax (see discussion 4.5. Corydalidae vs. Sialidae). In the fossil this could represent the plesiomorphic state in Megaloptera, as representatives of Corydalidae have a prothorax longer than wide. However, this seems to be unlikely as a wider than long prothorax is not only present in most extant representatives of Sialidae but also in the Jurassic fossil *Sharasialis fusiformis* [14], which is not only older than the newly described morphotype, but shows possibly more plesiomorphies and has been discussed as the sister group to all extant representatives of Sialidae [80]. Hence, the longer prothorax is probably a convergence to the state in Corydalidae.

The mouthparts of the two specimens are similar to each other as well as to the mouthparts of extant representatives of Sialidae, at least in certain aspects. The labrum is forward projecting and bears setae (Figure 7D and Figure 9F), the appearance is similar to those of extant representatives of Sialidae [5] (figure 70), [36] (figures 1–4 and 9), [58] (figure 10A), [68] (figure 7B), [95] (figures 7B, 16, 25, 36, 45, 54, 65, 75, 86, 91 and 96A), [151] (figure 7B). The labrum of first stage larvae of Sialidae seems to differ by being more rounded (compare [36] (figures 18–21)). This indicates the fossil specimens being of a later stage, which is further supported by the overall large size (15.2 mm & 13.5 mm). The antennae of PED 2821 and BUB 5274 both consist of four elements each (Figure 7D and Figure 9F). Four antenna elements are typical for extant representatives of Sialidae, further supporting the two fossils being representatives of this group [60,96]. The maxillae of both specimens show a well recognisable palp and an unarticulated process, interpreted as endite (Figure 7D and Figure 9F,G). The maxillae are most similar to those of extant representatives of Sialidae (compare [5] (figure 70B), [58] (figure 10B), [82,95] (figure 8B)). Extant representatives of Sialidae have been reported to have two endites on the maxilla that have been interpreted as galea and lacinia [5,58,95]. There are however difficulties with the interpretation of the structures of the maxilla. First Beutel & Friedrich [58] and Pereira [95] interpret the more proximal endite as lacinia and the more distal one as galea; New & Theischinger [5] do it the opposite way. Second the origin of the more distal endite is not recognisable in most of the drawings [5,58], but in the specimens shown by Pereira [95] (figure 8A,B) and Piraonapicha [100] (figure 9E) it is clearly originating from the most proximal element of the palp of the maxilla. This contradicts the interpretation as galea as well as lacinia, as they are both directly originating from the stipes. Hence, we will use the neutral term endite to discuss the structure. In PED 2821 a possible second, more proximal endite can be seen (Figure 7D), but no further details are recognisable. Due to the preservation in BUB 5274, it cannot clearly be stated if the second endite is truly absent or not recognisable.

The labium of PED 2821 is not accessible due to preservation. Also the labium of BUB 5274 is hard to differentiate. Still the submentum, mentum, ligula, and the palps are recognisable (Figure 9F,G). In contrast to modern representatives of Sialidae the submentum seems to be shorter than the mentum (compare [5] (figure 70B), [58] (figure 10B), [82] (figure 11), [95] (figure 8B)). The overall shape of the labium is, even with the different proportions of mentum and submentum, more similar to that of representatives of Sialidae than of representatives of Corydalidae. Extant representatives of Corydalidae have a submentum circumcising the mentum [58] (figure 2B), [151] (figure 6C).

The mandibles of BUB 5274 show clearly two teeth plus tip, as present in many extant larvae of Sialidae [5] (figure 68 (right)), [36] (figures 1–4 and 9), [58] (figure 10A), [68] (figure 7B), [70] (figure 23), [82] (figure 6), [95] (figures 16, 25, 36, 45, 54, 65, 75, 86, 91 and 96E), [100] (figure 9D), [151] (figure 7A). PED 2821 is more difficult to interpret. The mandibles are not bent outwards like in BUB 5274, but still well recognisable (Figure 7D,F). Both mandibles show clearly two teeth plus tip (Figure 7A), but the left mandible from the dorsal perspective (Figure 7D,F) seems to show an additional third proximal tooth (Figure 7F). This really differs from the observation on extant larvae of Sialidae as usually having two teeth plus tip. The only mandible that shows an additional third possible tooth on the mandible is illustrated by Gepp [41]. Also, in species of *Ilyobius* a so-called “dente basal” [95] (p. 18 figure 8D) has been described additional to the two teeth plus tip. However, this additional proximal tooth seems always rather small and not as prominent as the potential tooth of the fossil. This third tooth in the fossil is potentially an artefact of several structures laying above each other. Also, the potential tooth is not recognisable from the dorsal side. A mandible of an early representative of Sialidae is not necessarily restricted to only two teeth. Representatives of Corydalidae have been described with up to four teeth [58,151]. Hence, an early representative of Sialidae with three teeth could indicate that more teeth are part of the ground pattern in Megaloptera and the mandible with two prominent teeth is an autapomorphy for Sialidae. However, to further substantiate this a specimen with clearly three teeth plus tip would be needed. Additionally, the mandibles of both specimens are rather curved (Figure 7D and Figure 9F). This is untypical for most extant representatives of Sialidae.

The legs bearing numerous setae especially at the femur (Figure 7E and Figure 9D) as well as the relatively broad appearance of the femur of BUB 5274 (Figure 9A,D) is similar to those of extant representatives of Sialidae (compare [5] (figure 68 (right)), [48] (figure 29A), [151] (figure 7C–E), [95] (e.g., figures 7B, 16, 25, 36, 45, 54, 65, 75, 86, 91 and 96D,F,H). Coleoptera as a probable alternative interpretation to Sialidae seems unlikely, as this combination of characters (terminal filament, lateral gills, two claws) is, to our knowledge, not known in any beetle larva so far.

*Identity of specimen PED 1556 and morphotype 4 [9]*

Specimen PED 1556 exhibits a morphology distinct from that of the other newly described fossils. This morphology is similar to the “morphotype 4” described by Baranov et al. [9] and shares certain characters with larvae of Megaloptera. This includes the presence of lateral filamentous structures (e.g., Sialis [119]), large, toothed, forward-protruding mandibles, and antennae with four elements [60]. Prokin & Bashkuev [12] treated the morphotyp 4 as representative of Chauliodinae. Fossils of the morphotype 4, being superficially similar to extant megalopteran larvae, were already discussed by Baranov et al. [9], as possible beetle larvae. However, due to the superior preservation of the new specimen relative to the material in Baranov et al. [9], numerous additional larval characters can be discerned. These include: a head capsule with a prominent, lyriform moulting suture (Figure 5D); dorsal body surfaces with distinct structural modifications; a prominent digitiform sensillum at the antenna (Figure 5E); legs composed of five elements, the terminal one being a single claw (Figure 5C,G); rigid-appearing dorso-lateral abdominal filamentous structures composed of several parts (Figure 5H); paired, balloon-like structures (possible gills) on abdomen segments 1 and 8 (Figure 5A,H); and elongated structure (possible urogomphi) at the trunk end (Figure 5A,H). These morphological features can be found in representatives of one extant beetle lineage—Myxophaga. In particular, balloon-like tracheal gills on these specific segments are, so far, known exclusively from larvae of Myxophaga [119,153]. Larvae of Myxophaga are small, aquatic, and those with such balloon-like gills are typically associated with a habitat of a thin water layer on stones (hygropetric; [154]).

Not all characters of the new fossil, however, correspond to those known from extant myxophagan larvae. For example, the antennae appear to consist of four elements, a condition unknown in the so far described larvae of the group, although reduction in number of elements of the antennae is generally interpreted as derived in larvae of Myxophaga. More important: the mandibles of the new fossil do not resemble mandibles of known extant larvae. The extant larvae of Myxophaga have specialised semi-entognathous mouthparts, where mandibles and an endite of the maxilla are enclosed within a specialised structure derived from the labrum and the head capsule [154]. This state is a derived feature, possibly a result of miniaturisation and adaptation to algae-feeding lifestyle [154]. The mandibles in the fossil are freely articulated and not within a kind of pouch.

Furthermore, no modern larva of Myxophaga combines all the features present in the fossil; instead, these traits are distributed across representatives of different ingroups with extant representatives (e.g., Torridincolidae, Sphaeriusidae, Hydroschaphidae). Possible interpretations are that this type of fossil represents: (1) an extinct lineage within Myxophaga; (2) a larva of a lineage within Myxophaga of which the (fossil) larvae are not yet known, e.g., Lepiceridae [155]; (3) the sister group to Myxophaga, therefore retaining a plesiomorphic type of mouthparts.

In Kachin amber few representatives of Myxophaga, namely of the ingroups Sphaeriusidae [156,157] and Lepiceridae [158] have been reported. These specimens are all adults; so far no larva of Myxophaga has been reported. The morphotype 4 could represent the larva of one of the described species more likely that of Lepiceridae. If the larval morphotype is indeed of the group Lepiceridae, it clearly differs from the possible larva described by Lawrence et al. [159]. It can not be said for sure, if one of the two larvae, both or none is a representative of Lepiceridae. It is possible that previously discussed possibility 1 or 3 for the new fossil morphology is true or that, for example, the initial morphology changed over 100 million years of evolution. For further substantiation of this assumptions pupa stages associated with larvae and adults will be necessary.

Myxophagan larvae are so far rare in the fossil record. So far only a single candidate has been reported [160]. However, the identity of this fossil as representative of Myxophaga has already been questioned [161]. Therefore, morphotype 4 could represent the first myxophagan beetle larva preserved in amber.

*Identity of specimen BUB 5196*

The specimen can be recognized as an adult representative of Megaloptera. Further it can be interpreted as a representative of Sialidae. The prothorax is wider than long (Figure 10F), the fourth element of the tarsus is bilobed, and the distal element of the tarsus bears two claws (Figure 10G,H) as described for extant representatives of Sialidae [80]. From Kachin amber so far one species has been described [86]. This species is also an ingroup of Sialidae, male and female have been described [86,89]. The overall morphology of the new specimen is similar to the already described specimen, especially concerning legs and wings (compare [86] (figures 1 and 2), [89] (figures 1 and 2)). A possible part of the genitalia is visible (Figure 10A), however it appears incomplete, making a comparison uninformative. No other character indicates that this specimen could represent a new species.

*Identity of specimen BUB 5220*

The specimen can be identified as a larva of Gyrinidae. The overall morphology is similar to specimens of Gyrinidae already described in the literature [97,110,162]. The morphology of the mouthparts (Figure 11C–E), two claws on the distal end of the tarsus (Figure 11G), lateral gills (Figure 11A,B,F) and four hook-shaped structures at the terminal end (Figure 11H) indicate that the specimen is a representative of Gyrinidae [121,147,163].

### 4.2. Shape Analyses over Time

**Figure 13:**
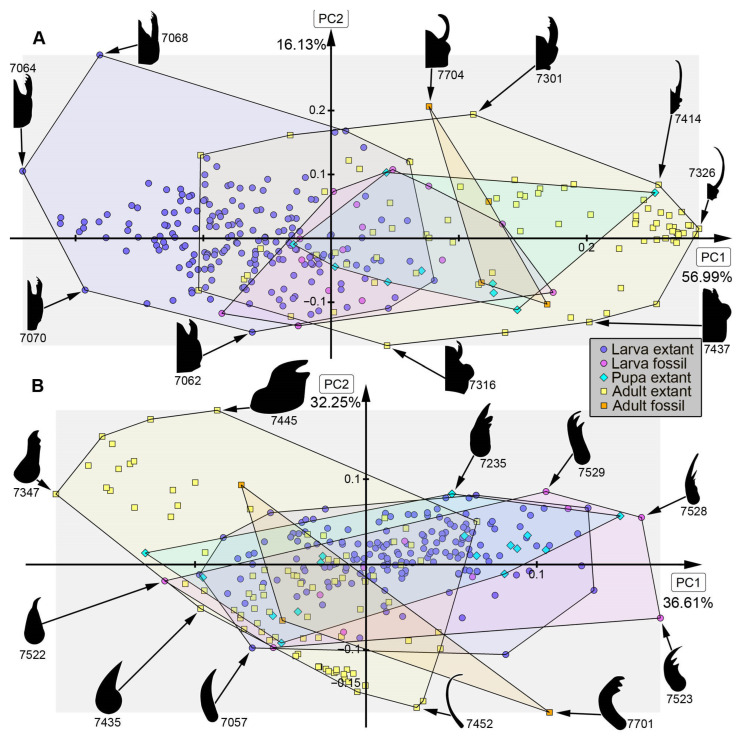
Scatter plots of principle component analyses of megalopteran & megalopteran-like specimens without Gyrinidae. PC2 plotted over PC1. Hulls show occupied area of morphospace of each group. (**A**) Analysis of head capsule and mandible (01). (**B**) Analysis of mandible (02).

**Figure 14:**
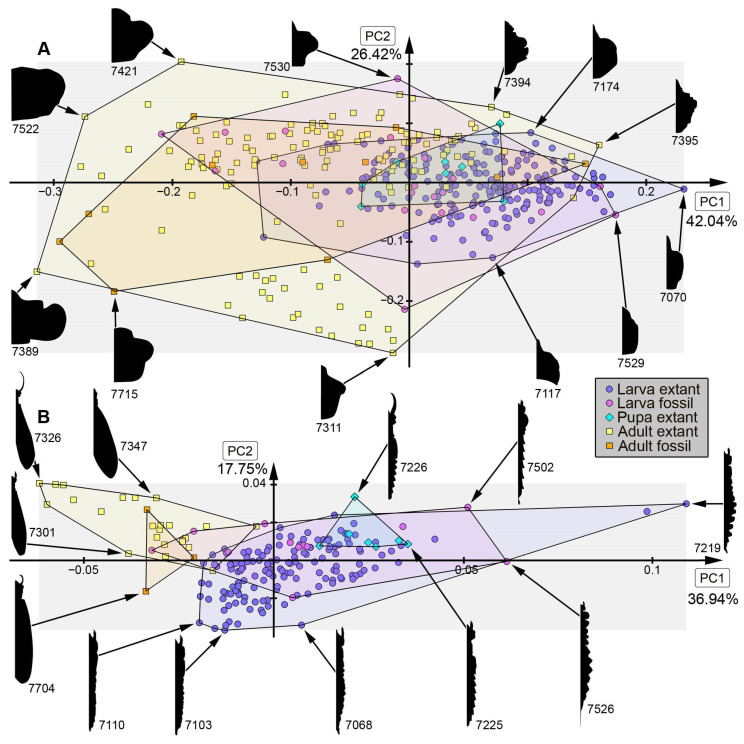
Scatter plots of principle component analyses of megalopteran & megalopteran-like specimens without Gyrinidae. PC2 plotted over PC1. Hulls show occupied area of morphospace of each group. (**A**) Analysis of head capsule (03). (**B**) Analysis of full body including the mandible (04).

**Figure 15:**
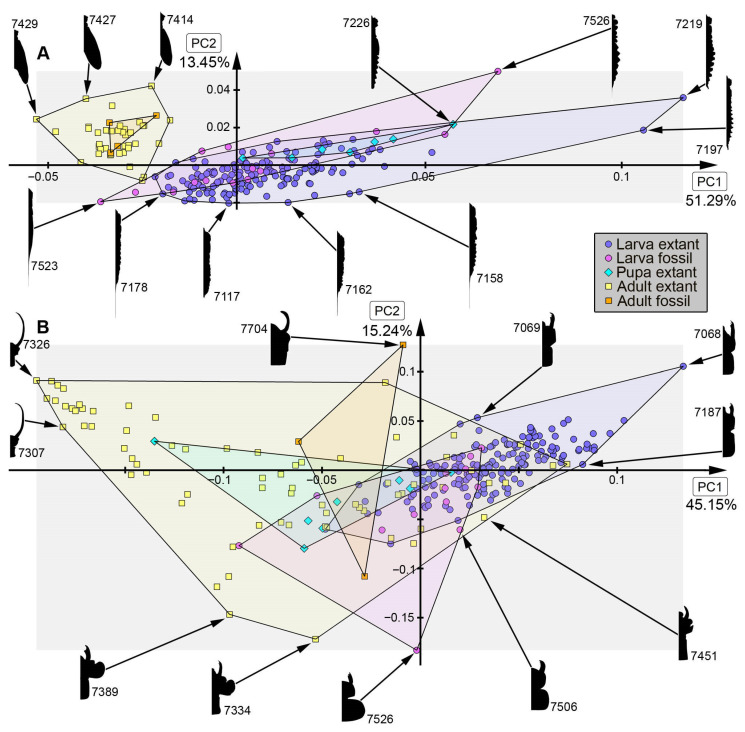
Scatter plots of principle component analyses of megalopteran & megalopteran-like specimens without Gyrinidae. PC2 plotted over PC1. Hulls show occupied area of morphospace of each group. (**A**) Analysis of full body without mandible (05). (**B**) Analysis of head capsule, prothorax and mandible (06).

**Figure 16:**
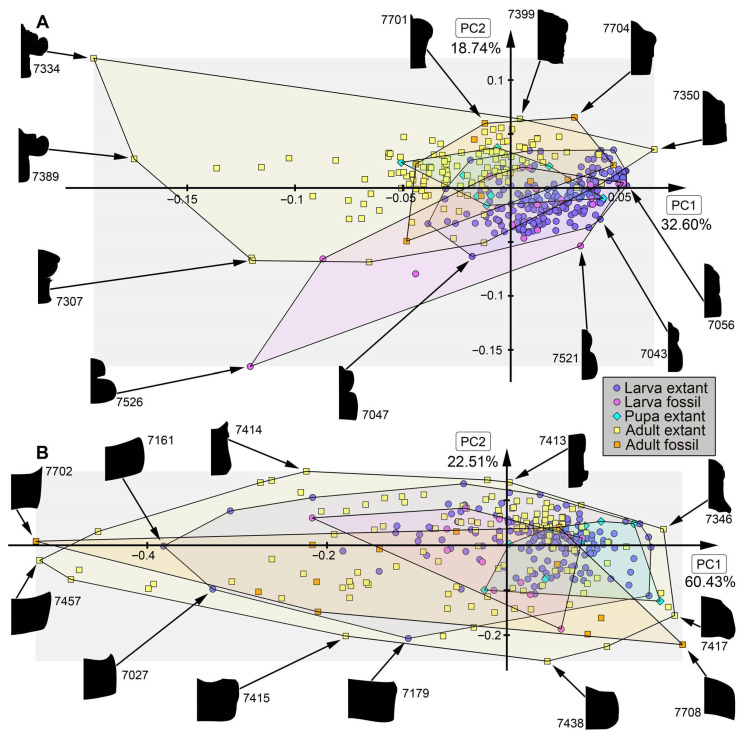
Scatter plots of principle component analyses of megalopteran & megalopteran-like specimens without Gyrinidae. PC2 plotted over PC1. Hulls show occupied area of morphospace of of each group. (**A**) Analysis of head capsule and prothorax (07). (**B**) Analysis of prothorax (08).

**Figure 17:**
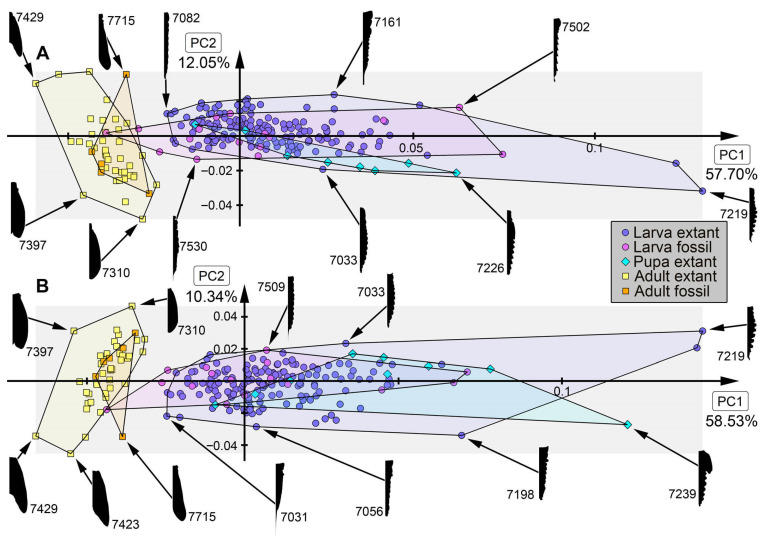
Scatter plots of principle component analyses of megalopteran & megalopteran-like specimens without Gyrinidae. PC2 plotted over PC1. Hulls show occupied area of morphospace of each group. (**A**) Analysis of trunk including the prothorax (09). (**B**) Analysis of trunk without prothorax (10).

**Figure 18:**
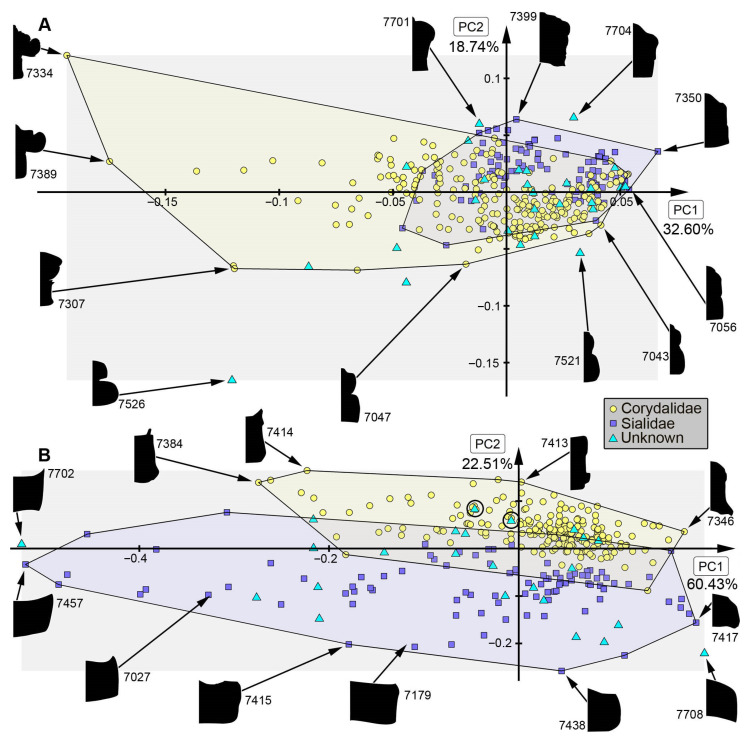
Scatter plots of principle component analyses of megalopteran & megalopteran-like specimens without Gyrinidae. Same analyses as in Figure 16, but different groups are marked. Hulls of Corydalidae and Sialidae show occupied area of morphospace of each group. Unknown specimens without hull. (**A**) Analysis of head capsule and prothorax (07). (**B**) Analysis of prothorax (08), black circles indicate position of PED 2821 (7523) and BUB 5274 (7529).

In most analyses fossil specimens occupy only an area within the occupied area of extant specimens, indicating no or only small losses in morphologies in most compared structures. The small sample size of megalopteran fossils must be taken into account, however, and might lead to an underrepresentation of the morphological diversity in the past. In some analyses, e.g., for mandible shape, some fossils stand out due a unique morphology that is not present in the extant representatives (Figure 13B: #7523, #7528, #7529, #7701). This unique morphology can be mostly characterized by highly curved mandibles with two protruding teeth in addition to the tip. The dentition of this morphotype appears highly similar to the mandible morphology in most extant representatives of Sialidae, but with the difference that there is more curvature in the fossils. These differences could indicate a different feeding strategy in this extinct morphotype compared to the feeding strategies used by extant representatives of Sialidae. Especially in the anterior body regions such as the head and head with mandibles, adult megalopterans show many unique morphologies that are not present in the fossil record (compare Figure 13A, Figure 14A, Figure 15B and Figure 16A), but also the trunk without the head capsule (Figure 17A,B). Also in the analysis of the head and prothorax a large area of the morphospace is occupied by different adult extant representatives of Corydalidae (Figure 16A and Figure 18A).

### 4.3. Discussion of Trialarva Coburgensis

**Figure 19:**
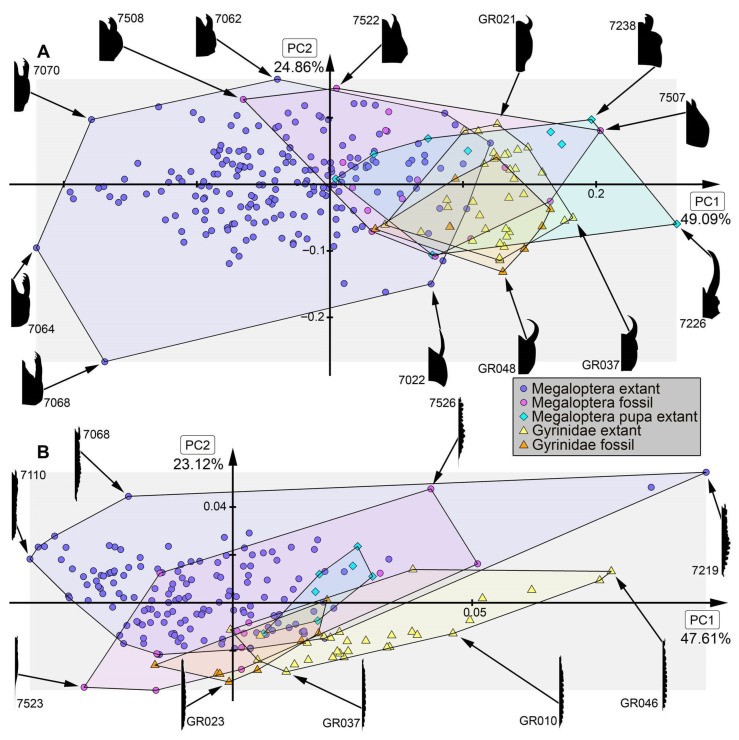
Scatter plots of principle component analyses of megalopteran & megalopteran-like larvae with Gyrinidae larvae. PC2 plotted over PC1. Hulls show occupied area of morphospace of each group. (**A**) Analysis of head capsule and mandible (01). (**B**) Analysis of full body including the mandible (04).

The obscure fossil *Trialarva coburgensis* plots as a strong outlier in some analyses, mostly due to the strong distinction of the segments along the lateral edges of the body (Figure 14B, Figure 15A,B, Figure 16A, Figure 18A and Figure 19B: #7526). The outlier position in some analyses indicates that if *T. coburgensis* is indeed a megalopteran, it has a rather unusual morphology. Due to its age, *T. coburgensis* could also indicate that the ancestral morphology of megalopteran larvae was different from later fossil and extant representatives. Prokin & Bashkuev [22] argued against *T. coburgensis* as a representative of Megaloptera based on a small head, narrower than the pronotum, the lack of numerous sutures of head capsule, the lack of distinct subdivision of the supposed gills, and the presence of similar-sized claws.

However, these arguments are relatively weak. First, a narrower head than the pronotum as a character state is present indeed in some representatives of Chauliodinae (*Protochauliodes aridus* [36]) and in the fossil *Cretochaulus lacustris* [15]. Second, the anterior region of *T. coburgensis* is not preserved well enough to give clear indication for the presence or absence of certain sutures, especially not of the clypeo-labral suture. Also, the lack of visible subdivision of the lateral filamentous structures is another weak argument, as the subdivision of the lateral filamentous structures is only strongly expressed in representatives of Sialidae [5] and often not clearly discernible even in highly resolved images of extant larvae of Corydalidae (compare e.g., [93]). Lastly as most of the walking legs are not preserved in *T. coburgensis* and the one that is present is also not well preserved, detailed interpretations such as the relation of the claw lengths to each other cannot be made as an objective observation. Furthermore, the point of unequal length of the claws on the walking legs is not strongly expressed in some representatives of Megaloptera and can also appear rather equal in size, depending on the angle of observation and also species (compare e.g., [93,96]). The long terminal unpaired filamentous structure of *T. coburgensis* can, however, be interpreted as a terminal filament corresponding to that in representatives of Sialidae. Another character suggested by Prokin & Bashkuev [22] supporting a coleopteran identity of *T. coburgensis* were structures supposed to be spiracles. However, the structures supposed to be tracheae (leading to the presumed spiracles) are rather massive. Additionally taking into account their striation pattern, these structures may represent muscles instead of tracheae and spiracles. We want to emphasize that it is also not particularly clear that *T. coburgensis* is a megalopteran, but the same is true for the previous interpretation as a representative of Coleoptera.

### 4.4. Ontogeny

The most distinct separation between larvae and adults can be observed in the analyses including the trunk (Figure 14B, Figure 15A and Figure 17A,B). This is due to the presence of the wings causing the shape appearing continuous compared to the larvae, including the pupae, which show a distinct separation of the segments along the abdomen. There are still two fossil larvae that plot together with the adults (Figure 14B, Figure 15A and Figure 17A,B, #7523: PED 2821 and data set #7501). This signal is most likely a result of the preservation, especially PED 2821 appears already partly dissolved (Figure 7A,C) and the separations of the segments cannot be well recognized. Also, the adult specimen plotting closest to the larvae (Figure 14B, Figure 15A and Figure 17A,B; #7424: [95] (figure 11)) is probably one that is also not well preserved, i.e., has deformed wings. The shape of the trunk appears less continuous than in the other adults. All pupae plot simply within the other larvae (Figure 15A and Figure 17A,B). This fits the expectations, as pupae are factually larvae [164] without fully developed wings and still have largely visible separations of the abdomen segments. Only when the mandible is included, one pupa plots outside the other larvae (Figure 14B, #7226: [71] (figure 3C)). This is a male representative of Corydalidae having a large, tusk-like mandible, due to sexual dimorphism. Here an adult character is already present in the (larval) stage of the pupa.

Concerning the analyses including the mandible, but without the trunk, the pupae plot more intermediate, between the adults and other larvae (Figure 13A,B and Figure 15B). Some pupae are even only plotting with the adults and not with the other larvae; again this is mainly due to the male pupae already developing the large, tusk-like mandibles. In general the large mandibles of the males expand the occupied area in the morphospace of all analyses including that of the mandible (Figure 13A,B, Figure 14B and Figure 15B). It is important to note that this does not reflect ecological diversity (e.g., feeding), because the mandibles of these males are only used for male-male competition and are not used for feeding anymore [60]. Also, the relatively short mandibles of certain representatives of Sialidae (Figure 13B, e.g., #7445: [80] (figure 7D)) expand the occupied morphospace of the adults, however these are non-feeding as well [35,80]. Generally, adult male megalopterans do not feed at all, only some adult females were observed feeding on fruit or nectar [35,60,80]. This phenomenon shows that morphological diversity of adults does not necessarily reflect ecological diversity. Larvae are therefore better proxies to reconstruct ecosystems based on interactions between the morphology of animals with their environment (for example in fossils). Hence, for the interpretation of the mandible diversity in adults we need to conclude, that it only has a minor known value for ecological interactions and interpretations. The larval mandibles expand the occupied morphospace over the one of the adults, especially by the presence and morphology of teeth (Figure 13B). This reflects the predatory lifestyle of the larvae. Also concerning the head capsule with mandible, the larvae extend the occupied morphospace. This expansion is especially due to larvae of Corydalidae with a prominent labrum and prominent mandibles (Figure 13A, e.g., #7064, #7068, #7070).

When the head capsule and the prothorax are considered, the adults occupy a larger area of the morphospace than the larvae (Figure 14A and Figure 16B). In the head capsule this seems to be driven by the compound eyes (Figure 14A, e.g., #7421: [94] (figure 3A)) or by the “cheeks” in some species of *Platyneuromus* (Figure 14A, e.g., #7389: [75] (figure 1C)). Yet, there are some larvae with a long labrum (Figure 14A, e.g., #7070), which is not present in adults. This reflects the different requirements on larvae (feeding) and adults (mating). Also, in analyses 06 (mandible, head capsule and prothorax, Figure 15B) and 07 (head capsule and prothorax, Figure 16A) these aspects impact the occupied areas of the morphospace. If the head capsule and the prothorax are considered, some larvae show a broader prothorax, compared to the head capsule (Figure 16A, e.g., #7043: [9] (figure 10C)), while the prothorax of adults is often smaller compared to the head capsule (Figure 16A, e.g., #7334: [42] (figure 2), #7399: [101] (figure 1B)). If only the prothorax is considered, the occupied area in the morphospace of the larvae is completely covered by that of the adults (Figure 16B). However, it seems not to be a large difference and the occupied areas of the larvae is only slightly smaller. Only in the most negative area in PC1 direction exclusively adults are present. This is a morphology of a relatively short prothorax with a concave anterior part enclosing the head capsule.

No stage of the larval phase was outstanding. The stage of the pupa already contains characters that are present in adults but not in other stages of the larval phase (Figure 13A,B, Figure 14B, Figure 15B and Figure 16A). Hence, the development seems to be rather typical for a group of Holometabola. For a more detailed comparison over the larval phase more first stage larvae from the extant fauna would be needed.

### 4.5. Corydalidae vs. Sialidae

Most analyses show a rather distinct separation of life stages (ontogenetic signal), and there is rarely a distinct separation between the two groups Sialidae and Corydalidae (phylogenetic signal). In the analysis of the head and prothorax and the analysis of the prothorax, however, such a phylogenetic signal can be observed (compare Figure 1B and Figure 18A). In the analysis of the head and prothorax a proportionally large area of the morphospace is occupied by representatives of Corydalidae due to some unique morphologies of the adults compared to the occupied area of adult representatives of Sialidae and megalopteran-like and megalopteran larvae. These proportional differences are not as strong in analyses with just the head capsule and seem to be related to the morphology of the prothorax. In the analysis of the prothorax this separation becomes even stronger, here quite distinct clusters can be observed, although there is still overlap. Representatives of Corydalidae show proportionally longer and sometimes slender morphologies, and representatives of Sialidae show proportionally shorter and wider morphologies. This signal could give indication for the group identity of difficult to interpret fossils with a preserved prothorax, which is especially often the case for the larvae. There is some overlap between the groups that is caused by representatives of Sialidae with an unusually long prothorax with one of the most extreme cases being the two newly described specimens PED 2821 (Figure 7) and BUB 5274 (Figure 9; see also discussion above). A similar condition can be seen in some extant representatives of Sialidae such as *Sialis lutaria* (#7022). A short prothorax (wider than long) has been discussed as an autapomorphy for the adults of Sialidae [80] and appears to also reflect the proportions in the immatures.

### 4.6. Larvae of Megaloptera and Gyrinidae

Overall, larvae of Gyrinidae share many aspects of morphology with those of Megaloptera, especially with larvae of Corydalidae. The most notable difference between the immatures of Megaloptera and Gyrinidae represents the morphology of the labrum: while the labrum is rather short in larvae of Gyrinidae, it is often very pronounced in larvae of Megaloptera. These superficial differences are also reflected in both analyses, the head with mandibles and the whole body. In the analysis of the head and mandible (Figure 19A) both groups largely overlap, but also occupy unique areas of the morphospace, mostly due to their labrum morphology in relation to the mandible length. Additionally, the curvature of the mandibles and the presence of teeth on the mandibles separates both groups to some extent. It must be noted that some representatives of Sialidae were included in the analysis with missing information on the dentition (e.g., #7022, Figure 19A). These specimens have been included as the mandible length is still accessible and gives valuable information about the morphology, but the high similarity in comparison to larvae of Gyrinidae likely represents an artifact. Interestingly, in the analysis for the whole body the fossil representatives of both groups show a large overlap, while in both groups extant representatives additionally occupy large unique areas of the morphospace (Figure 19B). This could indicate that originally both groups were morphologically (and possibly ecologically) more similar to each other and then specialized in different ways after the Cretaceous.

## 5. Conclusions

Megalopteran larvae remain rare in the fossil record, but we can see now first signals. It should not be surprising that more fossils become available as sedimentary fossils, so far it is in fact surprising why there are so few of these larvae in comparison to those of mayflies, dragonflies, or true flies. Similar to their closer relatives Raphidioptera [165] and Neuroptera [166,167] we have first indications that we lost some specialised larvae that were present in the Cretaceous. We can also find signals of possible evolutionary interactions, as present in their relatives [168], with other groups with similar larvae, in this case Gyrinidae, but these remain weak. We could also identify one of the earlier reported problematic morphotypes as a possible rare type of beetle larva, namely a myxophagan.

## Data Availability

All data from this study are available in this paper and the associated papers.

## Supplementary Materials

The following supporting information can be downloaded at https://www.mdpi.com/article/10.3390/insects17020197/s1, Supplementary Table S1: list of the dataset; Supplementary File S1: results of the shape analyses. Figures in high resolution are available at https://doi.org/10.5281/zenodo.18431993.
