# Supplementary material for: New Fossil Megalopteran and Megalopteran-like Larvae, a First Extinct Larval Morphology of Megaloptera, and Possible Larvae of Myxophagan Beetles
Source: Insects. 2026 Feb 12;17(2):197. doi: 10.3390/insects17020197 (PMC12940974; doi:10.3390/insects17020197)
Supplement: Supplementary file 1 [file insects-17-00197-s001.zip › Supplement_Megaloptera_Stahlecker_etal/Suppl File S1.zip!/Suppl File S1/05/05_Megaloptera.pdf]

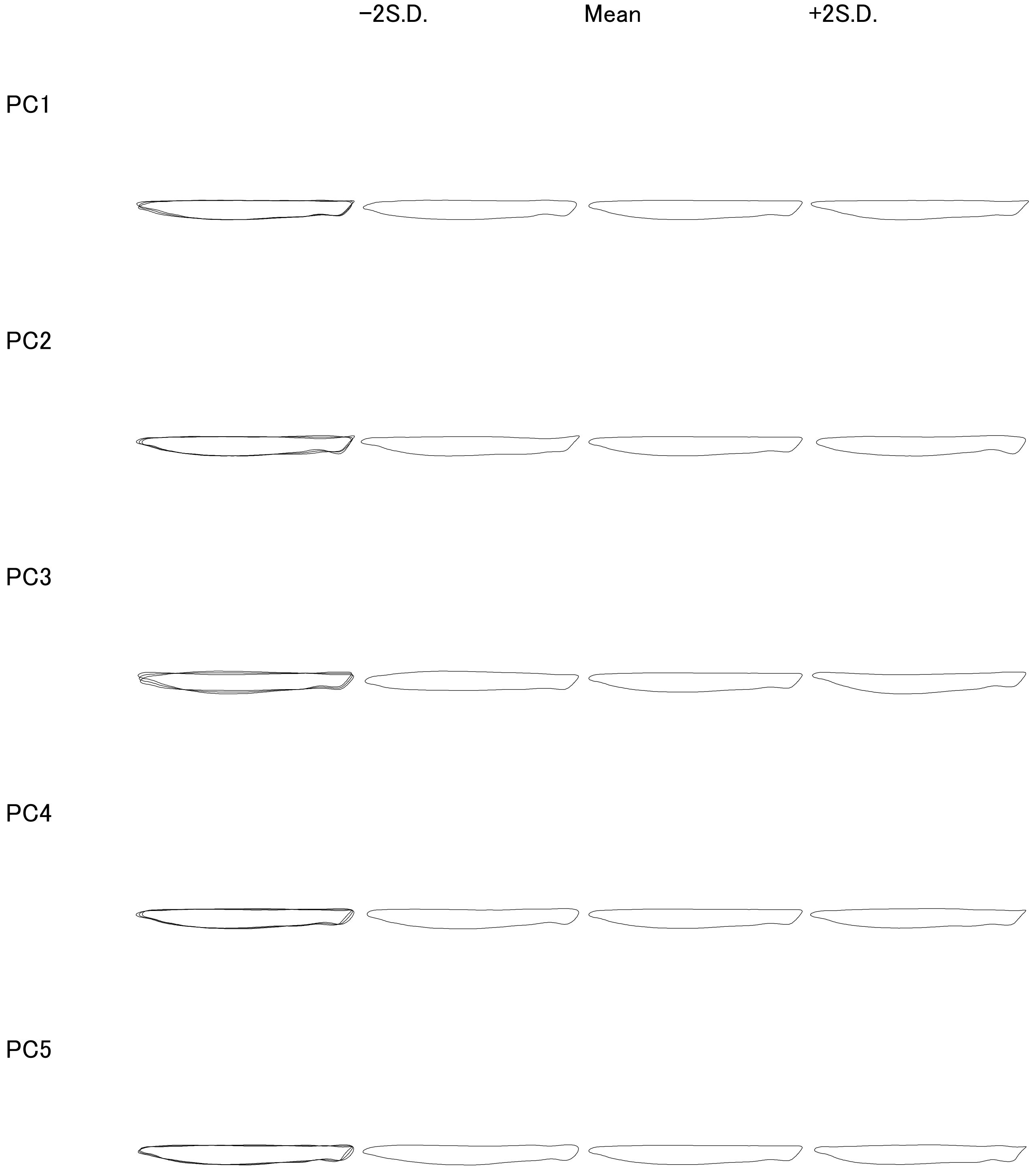
−2S.D.
Mean
+2S.D.
PC1
PC2
PC3
PC4
PC5

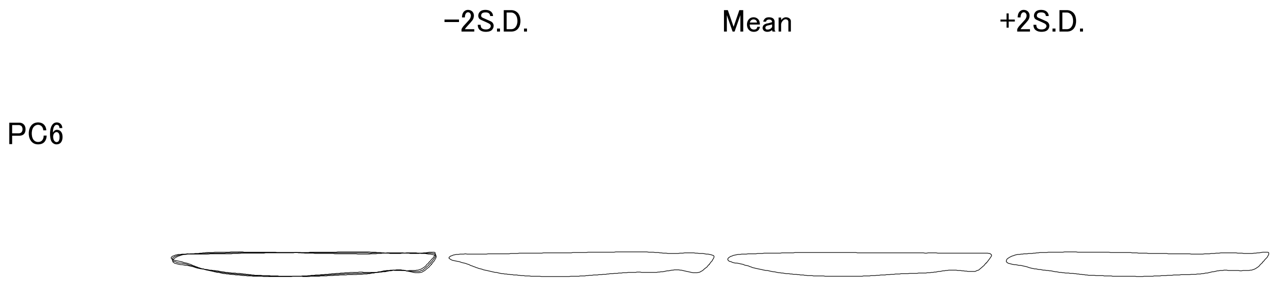
−2S.D.
Mean
+2S.D.
PC6
